# LARRPM restricts lung adenocarcinoma progression and M2 macrophage polarization through epigenetically regulating LINC00240 and CSF1

**DOI:** 10.1186/s11658-022-00376-y

**Published:** 2022-10-11

**Authors:** Yue Li, Chen Chen, Hai-lin Liu, Zhen-fa Zhang, Chang-li Wang

**Affiliations:** Department of Lung Cancer, Tianjin Medical University Cancer Institute and Hospital, National Clinical Research Center for Cancer, Key Laboratory of Cancer Prevention and Therapy, Tianjin’s Clinical Research Center for Cancer, Tianjin Lung Cancer Center, Tianjin, 300060 China

**Keywords:** Tumor-associated macrophage, Lung adenocarcinoma, Progression, DNA methylation, CSF1

## Abstract

**Background:**

Long non-coding RNAs (lncRNAs) are critical regulators in lung adenocarcinoma (LUAD). M2-type tumor-associated macrophages (TAMs) also play oncogenic roles in LUAD. However, the involvement of lncRNAs in TAM activation is still largely unknown.

**Methods:**

The expressions of LARRPM, LINC00240 and CSF1 were determined by RT-qPCR. The regulation of LINC00240 and CSF1 by LARRPM was investigated by RNA–protein pull-down, RNA immunoprecipitation, chromatin immunoprecipitation and bisulfite DNA sequencing. In vitro and in vivo gain- and loss-of-function assays were performed to investigate the roles of LARRPM.

**Results:**

The lncRNA LARRPM was expressed at low levels in LUAD tissues and cells. The low expression of LARRPM was correlated with advanced stage and poor survival of patients with LUAD. Functional experiments revealed that LARRPM suppressed LUAD cell proliferation, migration and invasion, and promoted apoptosis. LARRPM also repressed macrophage M2 polarization and infiltration. Taken together, LARRPM significantly restricted LUAD progression in vivo. Mechanistically, LARRPM bound and recruited DNA demethylase TET1 to the promoter of its anti-sense strand gene *LINC00240*, leading to a decrease in DNA methylation level of the *LINC00240* promoter and transcriptional activation of *LINC00240*. Functional rescue assays suggested that the lncRNA LINC00240 was responsible for the roles of LARRPM in the malignant behavior of LUAD cells. LARRPM decreased the binding of TET1 to the *CSF1* promoter, resulting in increased DNA methylation of the *CSF1* promoter and transcriptional repression of *CSF1*, which is responsible for the roles of LARRPM in macrophage M2 polarization and infiltration. The TAMs educated by LUAD cells exerted oncogenic roles, which was negatively regulated by LARRPM expressed in LUAD cells.

**Conclusions:**

LARRPM restricts LUAD progression through repressing both LUAD cell and macrophages. These data shed new insights into the regulation of LUAD progression by lncRNAs and provide data on the potential utility of LARRPM as a target for LUAD treatment.

**Supplementary Information:**

The online version contains supplementary material available at 10.1186/s11658-022-00376-y.

## Background

Although the incidence of lung cancer has been surpassed by that of breast cancer, lung cancer remains the leading cause of cancer death worldwide, with an estimated 2.2 million new cases and 1.8 million deaths in 2020 [1]. Lung adenocarcinoma (LUAD) has become the major histological subtype of lung cancer [2, 3]. Surgical resection is still the major treatment for patients wit LUAD [4]. However, for unresectable and recurrent LUAD, the prognosis is still very poor with currently available chemotherapy and molecule-targeted therapy [5]. More in-depth investigations of the mechanisms driving LUAD progression are urgently needed to develop more efficient treatment.

An increasing body of evidence is showing that the tumor microenvironment (TME) plays critical roles in LUAD [6]. In the TME, aberrant apoptosis of T cells is well known, which promotes the development of PD-1/PD-L1 antibodies for LUAD treatment [7]. In addition to T cells, tumor-associated macrophages (TAMs) are critical factors influencing LUAD by significantly promoting LUAD progression [8, 9]. Under specific local environments, monocytes/macrophages generally differentiate into the M1 or M2 subtype [10–12]. M1-polarized macrophages, also known as classical activated macrophages, routinely exert anti-cancerous effects [13, 14]. M2-polarized macrophages, also known as alternatively activated macrophage, routinely exert tumor-promoting effects [12, 15, 16]. Results from an increasing number of investigations suggest that TAMs resemble M2-polarized macrophages [17]. Given the critical roles of TAM in malignancies, many studies have been undertaken to dissect the mechanisms regulating TAM infiltration, polarization and functions [18–21].

Genome and transcriptome high-throughput sequencings have revealed that < 2% of the human genome encodes the information needed to make a protein while about 75% of the human genome encodes for RNAs [22]. Thus, most of RNA transcripts are non-coding RNAs (ncRNAs). Among these ncRNAs, long non-coding RNAs (lncRNAs) are a class of ncRNAs with more than 200 nucleotides in length [23–25]. Many studies have uncovered the critical roles of lncRNAs in many pathophysiological processes [26–31]. A lot of tumor-related lncRNAs have been identified that have oncogenic or tumor suppressive effects, including the regulation of cell proliferation, cell-cycle, apoptosis, motility, drug sensitivity, among others [32–39]. However, the involvement of lncRNAs in TAM activation is still unclear.

In the present study, we identified a LUAD-related lncRNA, LOC100270746, with the National Center for Biotechnology Information (NCBI) Reference Sequence Number of NR_026776.1. *LOC100270746* is located at chromosome 6p22.2 and at the anti-sense strand of *LINC00240*. We found that LOC100270746 modulated DNA methylation of the *LINC00240* promoter. Thus, LOC100270746 was named LINC00240 antisense RNA regulating promoter methylation (LARRPM). LARRPM is 954 nucleotides (nt) long and has a poly(A) tail. Two online in silico tools, namely Coding Potential Calculator 2 (CPC2) (http://cpc2.gao-lab.org/) and Coding Potential Assessment Tool (CPAT) (http://lilab.research.bcm.edu/), both indicated that LARRPM has no protein-encoding potential. The expression, clinical relevance, functions and mechanisms of action of LARRPM in LUAD were further investigated.

## Materials and methods

### Patient tissue samples

Seventy pairs of frozen LUAD tissues and adjacent lung tissues were acquired from patients with LUAD who had received surgery at our hospital. Written informed consent was obtained from all patients. Clinicopathological features of these 70 patients are provided in Additional file 1: Table S1.

This study was conducted in accordance with the Declaration of Helsinki and with the approval of the Ethics Committee of Tianjin Medical University Cancer Institute and Hospital (No. Ek2020183).

### Cell culture and reagents

Human bronchial epithelial cell 16HBE (Cat. SCC150) was acquired from Merck Millipore (MilliporeSigma, Burlington, MA, USA) and cultured in Airway Epithelial Cell Basal Media (American Type Culture Collection [ATCC], Manassas, VA, USA) supplemented with Bronchial/Tracheal Epithelial Cell Growth Kit components (ATCC). Human LUAD cells A549 (Cat. CCL­185), H1299 (Cat. CRL­5803), H1975 (Cat. CRL­5908), HCC827 (Cat. CRL-2868) and human monocyte line THP-1 (Cat. TIB-202) were obtained from the ATCC and cultured in F­12K (A549) or RPMI­1640 (H1299, H1975, HCC827, and THP-1) media (Invitrogen, Thermo Fisher Scientific, Waltham, MA, USA) supplemented with 10% fetal bovine serum (FBS; Invitrogen, Thermo Fisher Scientific). All cells were cultured in a humidified incubator at 37 °C and under 5% CO_2_ . These cells were authenticated by their short tandem repeat (STR) profiles and determined to be mycoplasma free. LUAD cells were treated with 0.2 µg/ml anti-CSF1 antibody (AF216; R&D Systems, Minneapolis, MN, USA) and THP-1 cells were treated with 100 ng/ml phorbol-12-myristate-13-acetate (PMA; Sigma-Aldrich, St. Louis, MO, USA).

### RNA extraction, reverse transcription and quantitative PCR

Total RNA was extracted using the TRIzol Reagent (Invitrogen, Thermo Fisher Scientific). Following quantification by the NanoDrop (Thermo Fisher Scientific), the RNA was subjected to reverse transcription (RT) to generate the first-strand complementary DNA (cDNA) with the M-MLV Reverse Transcriptase (Invitrogen, Thermo Fisher Scientific) and random primers. The cDNA was then subjected to quantitative PCR (qPCR) with the TB Green Premix Ex Taq II (TaKaRa, Tokyo, Japan) on the StepOnePlus Real-Time PCR System (Thermo Fisher Scientific). The primer sequences were as follows: 5′-ATTCATGGTGTCTCTACGCTG-3′ (sense) and 5′-TTTCCCCAAACTCTCCTCTC-3′ (anti-sense) for LARRPM; 5′-TATTCCTTGCCAACCCTCA-3′ (sense) and 5′-GCAGCCAGACAACTTTTTTC-3′ (anti-sense) for LINC00240; 5′-GATCTAGCACAGACCCTTCAC-3′ (sense) and 5′-CGACACCATCGTTACCTTGA-3′ (anti-sense) for MALAT1; 5′-GACTTTAAGGGTTACCTGGGTTG-3′ (sense) and 5′-TCACATGCGCCTTGATGTCTG-3′ (anti-sense) for IL10; 5'-TTTGATGTTGACGGACTG-3' (sense) and 5'-ATAGGCTTGTGATTACCC-3′ (anti-sense) for ARG1; 5′-TTTGTCAACTTGAGTCCCTTCAC-3′ (sense) and 5′-TCCCGCTACACTTGTTTTCAC-3′ (anti-sense) for CD163; 5′-TCCGGGTGCTGTTCTCCTA-3′ (sense) and 5′-CCAGTCTGTTTTTGATGGCACT-3′ (anti-sense) for CD206; 5′-ACCAGGTGGAGTTCAAGAC-3′ (sense) and 5′-CAATAGTCACTGCCCGAAT-3′ (anti-sense) for IL12; 5′-CCTCTCTCTAATCAGCCCTCTG-3′ (sense) and 5′-GAGGACCTGGGAGTAGATGAG-3′ (anti-sense) for TNF-α; 5′-TTCTGTGCCTGCTGCTCA-3′ (sense) and 5′-GGGACACTTGCTGCTGGT-3′ (anti-sense) for CCL2; 5′-AGTTCTCTGCATCACTTGCTG-3′ (sense) and 5′-CGGCTTCGCTTGGTTAGGAA-3′ (anti-sense) for CCL3; 5′-TCTGCGTGACTGTCCTGT-3′ (sense) and 5′-GGCTGCTGGTCTCATAGT-3′ (anti-sense) for CCL4; 5′-TGGCGAGCAGGAGTATCAC-3′ (sense) and 5′-AGGTCTCCATCTGACTGTCAAT-3′ (anti-sense) for CSF1; 5′-ACTCACCTCTTCAGAACGAATTG-3′ (sense) and 5′-CCATCTTTGGAAGGTTCAGGTTG-3′ (anti-sense) for IL6; 5′-ATGTAGCGGATAATGGAAC-3′ (sense) and 5′-ATGTATTGCTTTGCGTTGG-3′ (anti-sense) for IFN-γ; 5′-GAAACCCACAACGAAATCTATG-3′ (sense) and 5′-GCTGAGGTATCGCCAGGAAT-3′ (anti-sense) for TGF-β1; 5′-GGTCTCCTCTGACTTCAACA-3′ (sense) and 5′-GTGAGGGTCTCTCTCTTCCT-3′ (anti-sense) for glyceraldehyde 3-phosphate dehydrogenase (GAPDH). GAPDH was used as endogenous control.

### Plasmid construction and stable cell line generation

Full-length sequences of the lncRNA LARRPM were amplified by PCR with the primers 5′-CCCAAGCTTACAGGAACATCCGGCGT-3′ (sense) and 5′-CGGGATCCACACCTTAAATATATAGTTTT-3′ (anti-sense), which were further cloned into the* Hin*dIII and* Bam*HI sites of the pcDNA3.1(+) vector (Invitrogen, Thermo Fisher Scientific) to construct the LARRPM expression vector. LARRPM full-length sequences were also cloned into the* Hin*dIII and* Bam*HI sites of the pSPT19 vector (Roche, Basel, Switzerland) to construct the LARRPM in vitro transcription vector. LINC00240 full-length sequences were amplified by PCR with the primers 5′-GGAATTCGTAATCCTCCCAGGGATT-3′ (sense) and 5′-GCTCTAGATATCTTCAAAGCTCAAGGTCAC-3′ (anti-sense) and the sequences subsequently cloned into the* Eco*RI and* Xba*I sites of the pcDNA3.1(+) vector to construct the LINC00240 expression vector. To generate LUAD cells with LARRPM stable overexpression, the LARRPM expression plasmid or pcDNA3.1(+) vector was transfected into A549 and H1975 cells by Lipofectamine 3000 (Invitrogen, Thermo Fisher Scientific). The cells were then treated with 1000 µg/ml neomycin to select cells overexpressing LARRPM or control cells. To generate LUAD cells with LINC00240 stable overexpression, the LINC00240 expression vector or the pcDNA3.1(+) vector was transfected into A549 cells using Lipofectamine 3000. The cells were then treated with 1000 µg/ml neomycin to select LINC00240-overexpressing cells.

Two pairs of cDNA oligonucleotides targeting LARRPM and one pair of cDNA oligonucleotides targeting LINC00240 were generated and cloned into the GenePharma Supersilencing Vector pGLVU6/Puro (GenePharma, Shanghai, China), which were further used to generate lentivirus short hairpin RnAs (shRNAs). Scrambled non-targeting lentivirus shRNA was employed as the control. The shRNA oligonucleotide sequences were as follows: 5′-GATCCGGGATTTACCAAACGCATTCCTTCAAGAGAGGAATGCGTTTGGTAAATCCCTTTTTTG-3′ (sense) and 5′-AATTCAAAAAAGGGATTTACCAAACGCATTCCTCTCTTGAAGGAATGCGTTTGGTAAATCCCG-3′ (anti-sense) for shRNA-LARRPM-1; 5′-GATCCGGTGTCTCTACGCTGAGTTAATTCAAGAGATTAACTCAGCGTAGAGACACCTTTTTTG-3′ (sense) and 5′-AATTCAAAAAAGGTGTCTCTACGCTGAGTTAATCTCTTGAATTAACTCAGCGTAGAGACACCG-3′ (anti-sense) for shRNA-LARRPM-2; 5′-GATCCGCAGTGAGTTAGCTACCTTCTTTCAAGAGAAGAAGGTAGCTAACTCACTGCTTTTTTG-3′ (sense) and 5′-AATTCAAAAAAGCAGTGAGTTAGCTACCTTCTTCTCTTGAAAGAAGGTAGCTAACTCACTGCG-3′ (anti-sense) for shRNA-LINC00240; 5′-GATCCGTTCTCCGAACGTGTCACGTTTCAAGAGAACGTGACACGTTCGGAGAACTTTTTTG-3′ (sense) and 5′-AATTCAAAAAAGTTCTCCGAACGTGTCACGTTCTCTTGAAACGTGACACGTTCGGAGAACG-3′ (anti-sense) for the shRNA-control. To generate LUAD cells with LARRPM stable silencing, LARRPM specific or control lentivirus shRNAs were infected into HCC827 cells. The cells were then treated with 2 µg/ml puromycin to select the LARRPM silenced cells or the control cells. To generate LUAD cells with LINC00240 stable silencing and LARRPM stable overexpression, LINC00240-specific or control lentivirus shRNAs were infected into A549 cells overexpressing LARRPM. The cells were then treated with 2 µg/ml puromycin and 1000 µg/ml neomycin to select cells with silencing of the lncRNA LINC00240 and LARRPM overexpression.

### Cell proliferation, apoptosis, migration and invasion assays

Cell proliferation was assessed by the Cell Counting Kit-8 (CCK-8) and 5-ethynyl-2′-deoxyuridine (EdU) incorporation assays. The CCK-8 assay was carried out using the Cell Counting Kit-8 (Cat. CK04-11; Dojindo, Kumamoto, Japan), and the EdU incorporation assay was performed using the Cell-Light EdU Apollo567 In Vitro Kit (Cat. C10310-1; RiboBio, Guangzhou, China). Cell apoptosis was evaluated using the caspase-3 activity assay and terminal deoxynucleotidyl transferase (TdT)-mediated dUTP nick end labelling (TUNEL) assay. Caspase-3 activity assay was carried out using the Caspase 3 Activity Assay Kit (Cat. C1115; Beyotime, Shanghai, China). The TUNEL assay was carried out using the One Step TUNEL Apoptosis Assay Kit (Cat. C1086; Beyotime). Cell migration and invasion were evaluated by transwell migration and invasion assays, respectively, both of which were performed as previously described without or with precoated Matrigel [40].

### Animal experiments

Female BALB/C athymic nude mice (7–8 weeks old) were acquired from the Model Animal Research Center of Nanjing University (Nanjing, China) and fed under specific pathogen-free (SPF) conditions. The chosen LUAD cells were injected into the tail vein of the mice, and 6 weeks later the mice were sacrificed, following which the lungs were resected and stained with hematoxylin and eosin (HE) to evaluate the growth and metastasis of LUAD cells in the lungs. In vivo proliferation was evaluated by immunohistochemistry (IHC) assay using primary antibodies against Ki67 (ab15580, 1:200; Abcam, Cambridge, UK) or PCNA (ab29, 1:6000; Abcam) as previously described [41]. In vivo apoptosis was evaluated using the TUNEL assay with the In Situ Cell Death Detection Kit TMR red (Cat. 12156792910; Roche) following the manufacturer’s manual. Macrophage infiltration was evaluated using immunofluorescence (IF) staining with the primary antibody against the F4/80 antigen (#30325, 1:400; Cell Signaling Technology, Boston, MA, USA). The investigators performing the HE, IHC, IF and TUNEL assays were blinded to mice allocation. The animal experiments were approved by the Ethics Committee of Tianjin Medical University Cancer Institute and Hospital (No. Ek2020183).

### Isolation of cytoplasmic and nuclear RNA

Cytoplasmic and nuclear RNA were isolated from A549 cells using the PARIS Kit (Cat. AM1921; Invitrogen, Thermo Fisher Scientific). The isolated RNA was further subjected to RT-qPCR.

### RNA–protein pull-down and western blot

LARRPM was transcribed from the LARRPM in vitro transcription plasmid with T7 RNA polymerase (Roche). After purification, LARRPM was end-labeled with desthiobiotin by the RNA 3' End Desthiobiotinylation Kit (Pierce, Thermo Fisher Scientific). The desthiobiotinylated RNA was further subjected to RNA–protein pull-down reaction with the Magnetic RNA–Protein Pull-Down Kit (Pierce, Thermo Fisher Scientific). The enriched protein was measured by western blot using the primary antibodies against DNA demethylase TET1 (ab191698 1 µg/ml; Abcam) or GAPDH (60004-1-Ig, 1:10000; Proteintech, Chicago, IN, USA) as previously described [42].

### RNA immunoprecipitation

The RNA immunoprecipitation (RIP) assay was conducted using the EZ-Magna RIP Kit (MilliporeSigma) and primary antibody against TET1 (SAB2700730, 5 µg; Sigma-Aldrich). The enriched RNA was measured by RT-qPCR.

### Chromatin immunoprecipitation

The chromatin immunoprecipitation (ChIP) assay was conducted with the ChIP Kit (ab500; Abcam) and the primary antibody against TET1 (SAB2700730, 5 µg; Sigma-Aldrich). The enriched DNA was measured by qPCR with the primers 5′-TTCCAGGGCTGTTTCTCG-3′ (sense) and 5′-CACTTCCGTTCCCGCTTA-3′ (anti-sense) for the *LINC00240* promoter and the primers 5′-GATTTCCCATAAACCACAT-3′ (sense) and 5′-CCCAGGCAAACTTTCACT-3′ (anti-sense) for the *CSF1* promoter. Their respective distal regions were used as negative controls, and the primer sequences were as follows: 5′-TCAACTAATGGTGGAAAG-3′ (sense) and 5′-CCAATGTAATGGTGCTAA-3′ (anti-sense) for the negative control of the *LINC00240* promoter; 5′-ACAAGGGCATTCAGTCCA-3′ (sense) and 5′-CGTCAGAGCCAGAGCATC-3′ (anti-sense) for the negative control for the *CSF1* promoter.

### Quantitative analysis of DNA methylation and hydroxymethylation

DNA methylation was measured using the bisulfite DNA sequencing method. DNA was extracted with the TIANamp Genomic DNA Kit (TIANGEN, Beijing, China), followed by bisulfite treatment with the EZ DNA Methylation-Gold Kit (Zymo Research, Irvine, CA, USA). Modified genomic DNA was amplified by PCR with the primers 5′-AGTTTTTTTGGAAAGAAGTA-3′ (sense) and 5′-TCCTTACACAACCTACAC-3′ (anti-sense) for CpG43, and 5′-AGGGTTGGTTAGTGAGGTT-3′ (sense) and 5′-ATCATACAAACAACTAAATCC-3′ (anti-sense) for CpG82. The PCR products were gel-extracted, cloned into the pMD-19 T vector (Takara), transformed into* Escherichia coli* cells and sequenced. DNA hydroxymethylation was measured using the EpiMark 5-hmC Analysis Kit (New England Biolabs, Ipswich, MA, USA), followed by qPCR to detect DNA hydromethylation of CpG43 and CpG82 with the above-described premiers.

### IF staining

After co-culture with LUAD cells, the PMA-stimulated THP-1 cells were first incubated with primary antibodies against CD163 (ab87099, 5 µg/ml; Abcam) or CD206 (#91992, 1:200; Cell Signaling Technology Cell Signaling). The cells were then incubated with goat anti-rabbit IgG (Alexa Fluor 488; Invitrogen, Thermo Fisher Scientific), stained with DAPI and photographed on a Zeiss photomicroscope (Carl Zeiss, Oberkochen, Germany).

### Enzyme-linked immunosorbent assay

An enzyme-linked immubosorbent assay (ELISA) was performed to measure CSF1 concentrations in cell culture supernates of the indicated LUAD cells using the Human M-CSF ELISA Kit (Cat. DY216; R&D Systems).

### Statistical analysis

The GraphPad Prism version 6.0 software package (GraphPad Software, San Diego, CA, USA) was used to conduct all statistical analyses. Details on the statistical methods applied are shown in the figure and provided in the table captions. *P* < 0.05 was considered to indicate statistically significance.

## Results

### LARRPM was lowly expressed and a prognosis-related lncRNA in LUAD

**Fig. 1:**
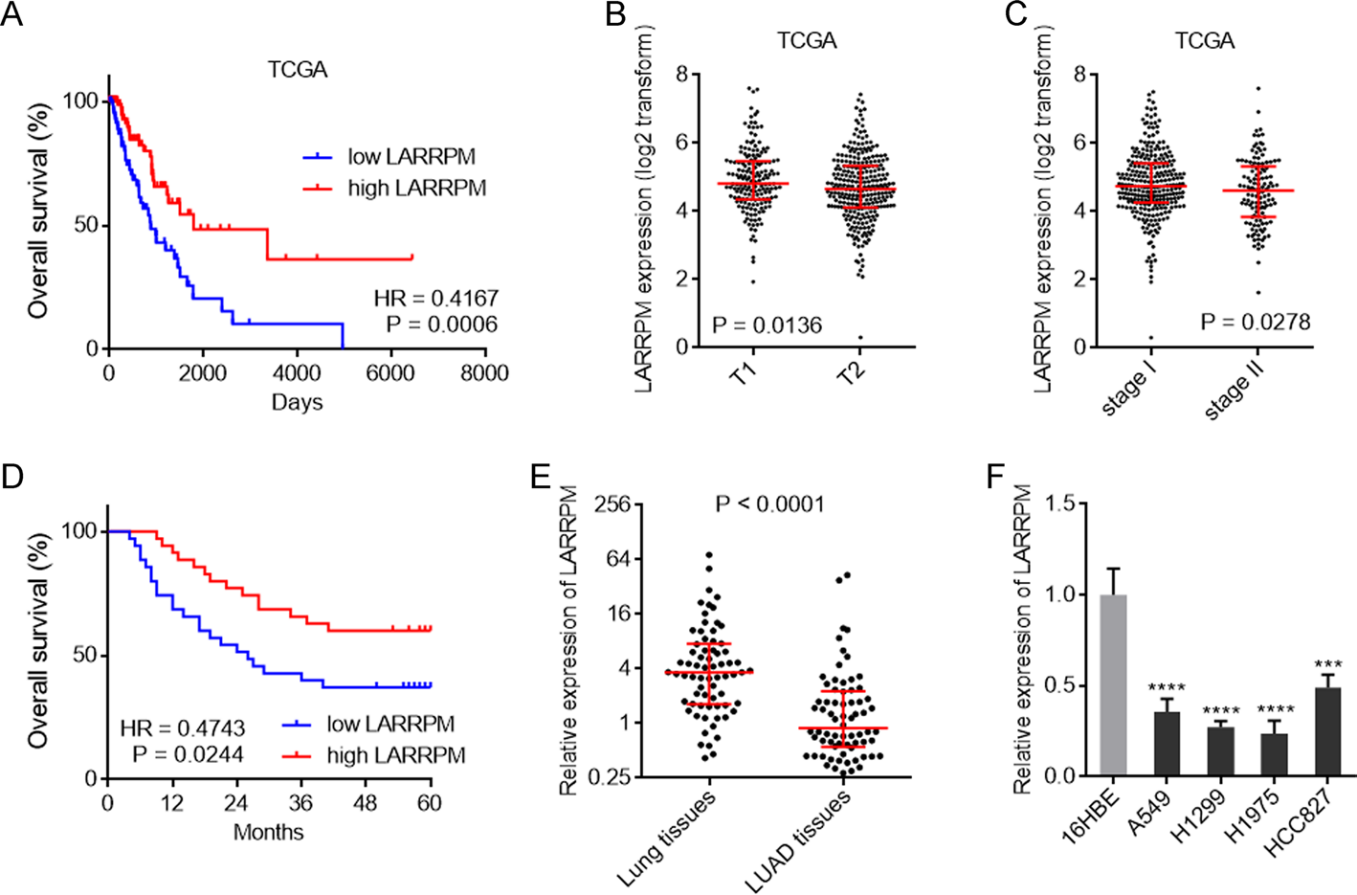
LARRPM was downregulated and associated with prognosis in LUAD. **a** Correlation between LARRPM expression and overall survival analyzed using the TCGA LUAD dataset. HR = 0.4167, *P* = 0.0006, log-rank test. *n* = 248, median LARRPM expression level was used as the cut-off. **b** LARRPM expression levels in T1-and T2-stage LUAD tissues analyzed using the TCGA dataset.* P* = 0.0136, Mann–Whitney test. **c** LARRPM expression levels in TNM stages-I and -I LUAD tissues analyzed using the TCGA dataset. *P* = 0.0278, Mann–Whitney test. **d** Correlation between LARRPM expression and overall survival analyzed using our LUAD cohort data. HR = 0.4743, *P* = 0.0244, log-rank test. *n* = 70, median LARRPM expression level was used as the cut-off. **e** LARRPM expression in 70 pairs of LUAD tissues and matched adjacent lung tissues was detected by qRT-PCR. *P* < 0.0001, Wilcoxon matched-pairs signed rank test. **f** LARRPM expression in 16HBE cells and LUAD cells A549, H1299, H1975, and HCC827 was detected by qRT-PCR. Results are shown as mean ± standard deviation (SD) based on three independent experiments. Asterisks indicate a significant difference at ***P < 0.001, ****P < 0.0001, by one-way analysis of variance (ANOVA) followed by Dunnett's multiple comparisons test.* 16HBE* Human bronchial epithelial cell line,* A549, H1299, H1975, HCC827* human LUAD cell lines,* HR* hazard ratio, * LARRPM* a LUAD-related long non-coding RNA,* LUAD* lung adenocarcinoma,* qRT-PCR* reverse-transcription quantitative PCR, * T1, T2* TNM stages T1, T2, *TCGA* The Cancer Genome Atlas

To analyze the clinical significance of LARRPM in LUAD, we first explored The Cancer Genome Atlas (TCGA) LUAD dataset using the online in silico tool R2 (https://hgserver1.amc.nl/cgi-bin/r2/main.cgi). The results showed that low LARRPM expression was correlated with poor overall survival in LUAD (Fig. 1a). The TCGA LUAD dataset also revealed that the expression of LARRPM was lower in tumor (T)2-stage LUAD tissues than in T1-stage LUAD tissues (Fig. 1b). The expression of LARRPM was also lower in stage II LUAD tissues than that in stage I LUAD tissues (Fig. 1c). To further confirm the clinical relevance of LARRPM in LUAD, we collected 70 pairs of LUAD and matched adjacent lung tissues. In our cohort, we also found that low expression of LARRPM in LUAD tissues was correlated with poor overall survival of patients with LUAD (Fig. 1d). Low expression of LARRPM was also correlated with large tumor size, local invasion and advanced TNM stages in our cohort (Additional file 1: Table S1). Furthermore, LARRPM was lowly expressed in LUAD tissues compared with that in noncancerous lung tissues (Fig. 1e). The expression of LARRPM in cells of human bronchial epithelial cell line 16HBE and in LUAD cells A549, H1299, H1975, and HCC827 was measured. The results revealed that, consistent with the expression pattern in tissues, LARRPM was also lowly expressed in LUAD cells compared with bronchial epithelial cell (Fig. 1f). These findings identified LARRPM as a lowly expressed and prognosis-related lncRNA in LUAD.

### LARRPM exerted tumor suppressive effects in LUAD in vitro

**Fig. 2:**
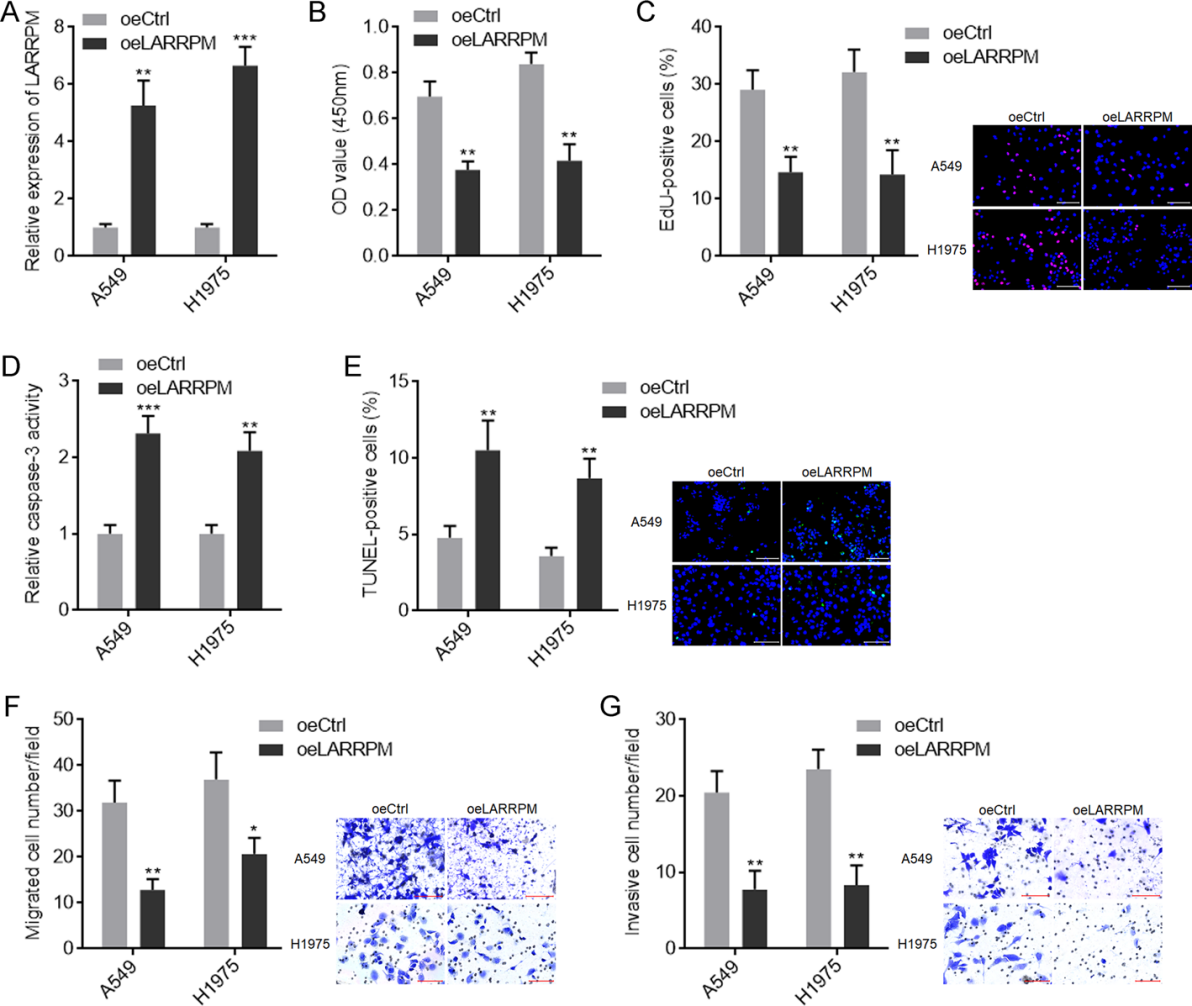
LARRPM repressed proliferation, induced apoptosis and inhibited migration and invasion of LUAD cells.** a** LARRPM expression in A549 and H1975 cells with LARRPM stable overexpression and A549 and H1975 control cells was detected by qRT-PCR. **b**, **c** Cell proliferation of A549 and H1975 cells with LARRPM stable overexpression and A549 and H1975 control cells was detected using CCK-8 assays (**b**) and EdU incorporation assays (**c**). Scale bar: 100 µm. Red color indicates EdU-positive cells. **d**, **e** Cell apoptosis of A549 and H1975 cells with LARRPM stable overexpression and A549 and H1975or control cells was detected using caspase-3 activity assays (**d**) and TUNEL assays (**e**). Scale bar = 100 µm. Green color indicated TUNEL-positive cells. **f**, **g** Cell migration and invasion of A549 and H1975 cells with LARRPM stable overexpression or A549 and H1975 control cells was detected using transwell migration assay (**f**) and transwell invasion assay (**g**). Scale bar = 100 µm. Results are shown as mean ± SD based on three independent experiments. Asterisks indicate a significant difference at *P < 0.05, **P < 0.01, ***P < 0.001, by the Student’s *t*-test.* CCK-8* Cell Counting Kit-8,* Ctrl* control,* EdU* 5-ethynyl-2′-deoxyuridine,* oe* overexpression,* Tunel* terminal deoxynucleotidyl transferase (TdT)-mediated dUTP nick end labeling

To investigate whether LARRPM plays active roles in LUAD progression, we constructed A549 and H1975 cells with LARRPM stable overexpression (Fig. 2a). CCK-8 assays showed that both A549 and H1975 cells with LARRPM overexpression had reduced cell viability (Fig. 2b). EdU incorporation assays revealed that both A549 and H1975 cells with LARRPM overexpression had reduced EdU-positive cells (Fig. 2c), indicating decreased cell proliferation. Caspase-3 activity assays revealed that both A549 and H1975 cells with LARRPM overexpression had increased caspase-3 activity (Fig. 2d), indicating increased cell apoptosis. TUNEL assays revealed that both A549 and H1975 cells with LARRPM overexpression had an increased number of TUNEL-positive cells (Fig. 2e), also indicating increased cell apoptosis. Transwell migration assays revealed that both A549 and H1975 cells with LARRPM overexpression had decreased cell migration (Fig. 2f). Transwell invasion assays revealed that both A549 and H1975 cells with LARRPM overexpression had decreased cell invasion (Fig. 2g).

To further investigate the roles of LARRPM in LUAD, we constructed HCC827 cells with LARRPM stable silencing via infection by two independent LARRPM-specific lentivirus shRNAs (Additional file 2: Figure S1A). The CCK-8 and EdU incorporation assays revealed that LARRPM silencing promoted cell proliferation (Additional file 2: Figure S1B, C). Caspase-3 activity assays and TUNEL assays showed that LARRPM silencing reduced cell apoptosis (Additional file 2: Fig. S1D, E). Transwell migration and invasion assays revealed that LARRPM silencing increased cell migration and invasion (Additional file 2: Figure S1F, G). Collectively, these findings suggested that LARRPM exerted tumor suppressive roles in LUAD in vitro.

### LARRPM restricted LUAD progression in vivo

**Fig. 3:**
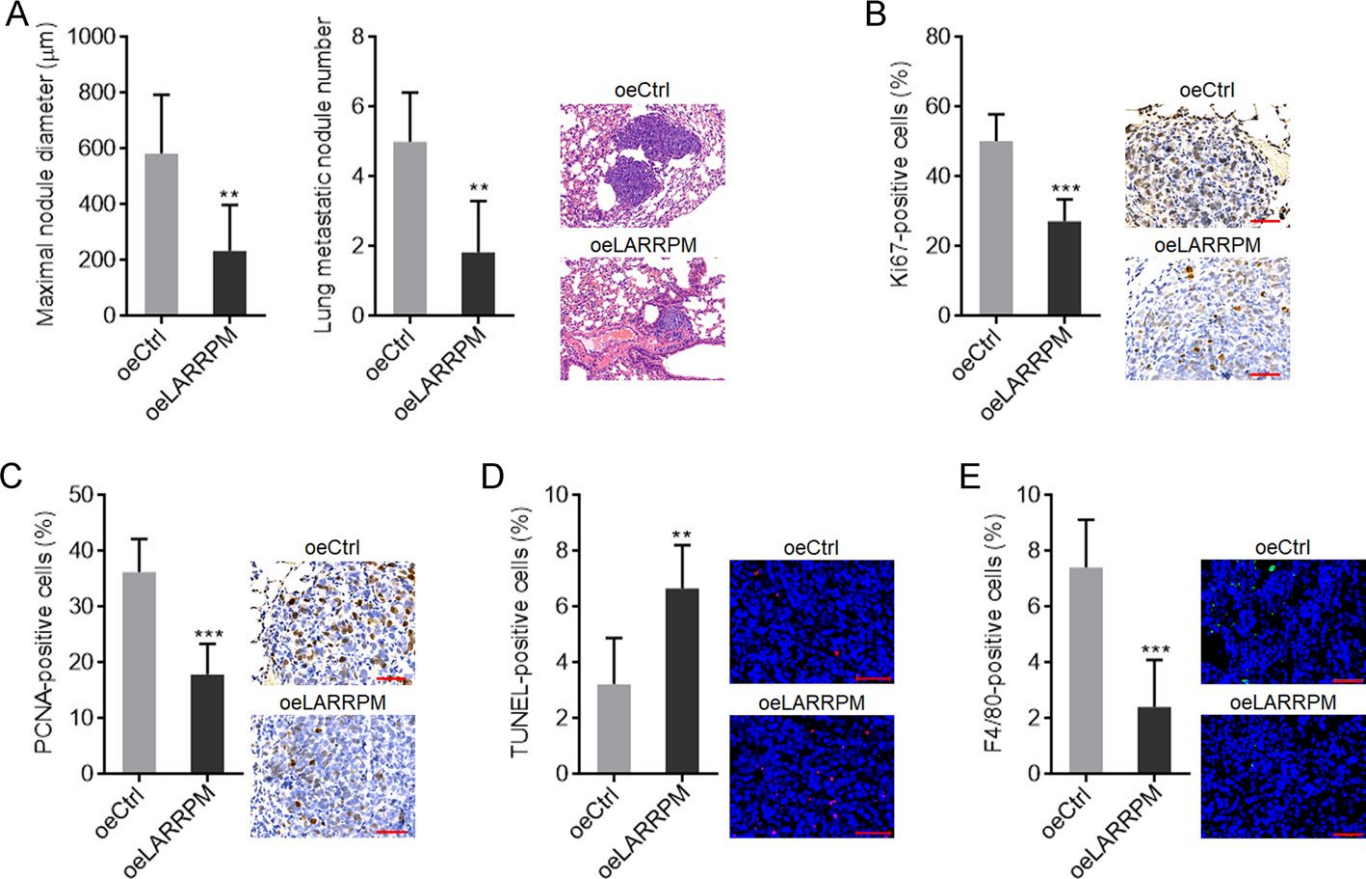
LARRPM repressed LUAD development in vivo. **a** A549 cells with LARRPM stable overexpression or A549 control cells were injected into tail vein of nude mice. Six weeks later, lung metastatic nodules were detected using hematoxyln and eosin (HE) staining. **b**, **c** Immunohistohemistry (IHC) staining of the antigens Ki67 (**b**) and PCNA (**c**) in lung metastatic nodules formed by A549 cells with LARRPM stable overexpression or A549 control cells. Scale bar: 50 µm. **d** TUNEL assays using lung metastatic nodules formed by A549 cells with LARRPM stable overexpression or A549 control cells. Scale bar = 50 µm. **e** Immunofluorescence (IF) staining of F4/80 antigen in lung metastatic nodules formed by A549 cells with LARRPM stable overexpression or A549 control cells control. Scale bar: 50 µm. Results are shown as the mean ± SD based on *n* = 6 mice in each group. Asterisks indicate a significant difference at **P < 0.01, ***P < 0.001 by the Student’s *t*-test

The roles of LARRPM in LUAD in vivo were then explored. A549 cells with LARRPM stable overexpression or control cells were injected into the tail vein of nude mice. Six weeks later, the lungs were resected and detected by HE staining. As shown in Fig. 3a, A549 cells with LARRPM overexpression formed significantly smaller and fewer lung metastatic nodules than the control A549 cells. IHC staining of proliferation markers Ki67 and PCNA revealed that the tumors formed by A549 cells with LARRPM overexpression had significantly fewer Ki67- and PCNA-positive cells (Fig. 3b, c), indicating that LARRPM inhibited cell proliferation in vivo. TUNEL assays revealed that the tumors formed by A549 cells with LARRPM overexpression had increased proportion of TUNEL-positive cells (Fig. 3d), indicating that LARRPM promoted cell apoptosis in vivo. In addition, IF staining of macrophage marker F4/80 revealed that the tumors formed by A549 cells with LARRPM overexpression had significantly reduced macrophage infiltration (Fig. 3e). Taken together, these findings suggested that LARRPM restricted LUAD progression via regulating both cancer cells and macrophages.

### LARRPM activated *LINC00240* expression via recruiting TET1

**Fig. 4:**
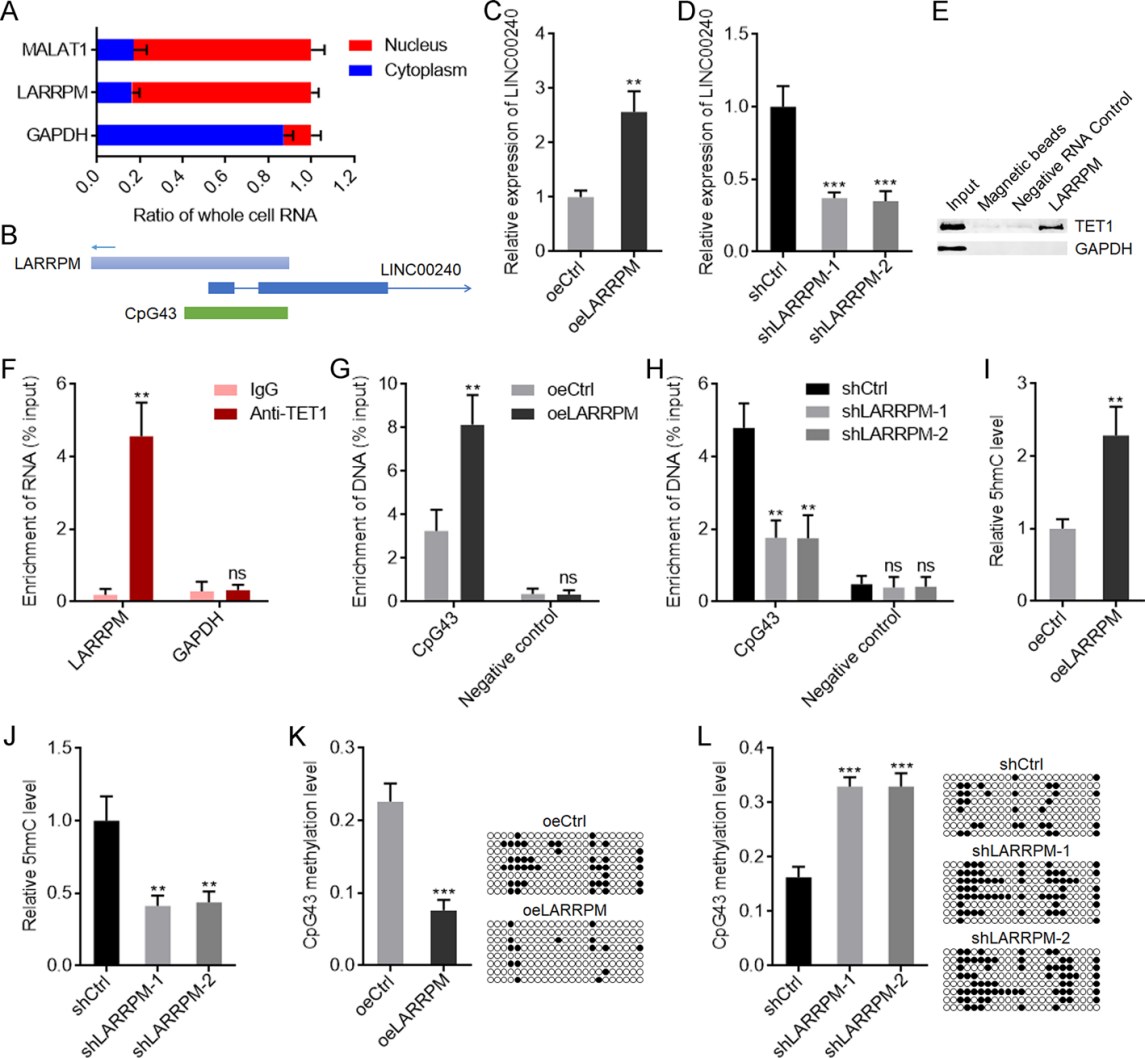
LARRPM upregulated LINC00240 expression via inducing DNA demethylation. **a** Subcellular localization of LARRPM in A549 cells was detected using subcellular fractionation followed by qRT-PCR. MALAT1 and GAPDH were used as nuclear and cytoplasmic controls, respectively. **b** The schematic diagram of the genomic localization of *LARRPM* and *LINC00240*. **c**, **d** LINC00240 expression in A549 cells with LARRPM overexpression (**c**) and HCC827 cells with LARRPM depletion (**d**) was detected by qRT-PCR. **e** RNA–protein pull-down assays followed by western blot were performed to detect the proteins bound by LARRPM. **f** RNA immunoprecipitation (RIP) assays followed by qRT-PCR were performed to detect the RNAs bound by TET1. **g**, **h** Chromatin immunoprecipitation (ChIP) assays followed by qPCR were performed in A549 cells with LARRPM overexpression (**g**) and HCC827 cells with LARRPM depletion (**h**) to detect the DNAs bound by TET1. **i**, **j** 5-Hydroxymethylcyctosine (5hmC) levels of CpG43 from A549 cells with LARRPM overexpression (**i**) and HCC827 cells with LARRPM depletion (**j**) were measured using the EpiMark 5-hmC Analysis Kit. **k, l** DNA methylation levels of CpG43 from A549 cells with LARRPM overexpression (**k**) and HCC827 cells with LARRPM depletion (**l**) were measured using bisulfate DNA sequencing. Results are shown as the mean ± SD based on three independent experiments. Asterisks indicate a significant difference at **P < 0.01, ***P < 0.001; ns, not significant, by Student’s *t*-test (**c**, **f**, **g**, **i**, **k**) or one-way ANOVA followed by Dunnett's multiple comparisons test (**d**, **h**, **j**, **l**).* GAPDH* Glyceraldehyde 3-phosphate dehydrogenase,* LINC00240* a long LUAD-related non-coding RNA, * MALATI* a long non-coding RNA, *TET1* a DNA demethylase

To dissect the mechanisms mediating the roles of LARRPM in LUAD, we first detected the subcellular localization of LARRPM in LUAD cells. Subcellular fractionation followed by qRT-PCR revealed that LARRPM was predominantly localized in the nucleus (Fig. 4a). The genomic localization of *LARRPM* was at the anti-sense strand of *LINC00240* (Fig. 4b), which has been reported to play various roles in different cancers [43–45]. We then studied the effect of LARRPM on LINC00240. The qRT-PCR results showed that LINC00240 increased when LARRPM was overexpressed (Fig. 4c) and decreased when LARRPM was silenced (Fig. 4d). Taking into account that the promoter of *LINC00240* contained the CpG island CpG43 (Fig. 4b), we hypothesized that LARRPM may modulate LINC00240 expression via DNA methylation. Intriguingly, the online in silico tool RNA–Protein Interaction Prediction (RPISeq) (http://pridb.gdcb.iastate.edu/RPISeq/index.html) predicted a strong interaction between LARRPM and DNA demethylase TET1, with an interaction probability score of 0.9. RNA–protein pull-down assays showed that TET1 was specifically enriched by LARRPM (Fig. 4e), and RIP assays showed that LARRPM was specifically enriched with TET1 antibody (Fig. 4f). Both the RNA–protein pull-down assays and RIP assays indicated interaction between LARRPM and TET1. ChIP assays were performed to investigate whether LARRPM modulates the binding of TET1 to the *LINC00240* promoter. The results showed that ectopic expression of LARRPM promoted the binding of TET1 to the *LINC00240* promoter, while depletion of LARRPM inhibited the binding of TET1 to the *LINC00240* promoter (Fig. 4g, h). In line with the binding of TET1 to the *LINC00240* promoter, ectopic expression of LARRPM increased the level of 5-hydroxymethylcyctosine (5hmC), while depletion of LARRPM decreased the level of 5hmC at the *LINC00240* promoter (Fig. 4i, j). The DNA methylation level of CpG43 was reduced when LARRPM was overexpressed, and increased when LARRPM was depleted (Fig. 4k, l). The TCGA LUAD dataset showed a significantly positive correlation between LINC00240 and LARRPM expression in LUAD tissues (Additional file 3: Figure S2A). In our own cohort, there was a significant positive correlation between LINC00240 and LARRPM expression in LUAD tissues (Additional file 3: Fig. S2B). We also randomly selected 20 LUAD tissues and measured 5hmC levels at the *LINC00240* promoter; the results showed that LUAD tissues with high LARRPM expression had higher 5hmC levels at the *LINC00240* promoter compared with LUAD tissues with low LARRPM expression (Additional file 3: Figure S2C). In these 20 LUAD tissues we further found that LUAD tissues with high LARRPM expression had a reduced DNA methylation level of CpG43 compared with LUAD tissues with low LARRPM expression (Additional file 3: Figure S2D). These clinical data supported the regulation of LINC00240 by LARRPM via TET1-mediated DNA demethylation. Collectively, these findings suggested that LARRPM bound to and recruited TET1 to *LINC00240* promoter, induced DNA demethylation of *LINC00240* promoter, and activated *LINC00240* expression.

### Depletion of LINC00240 reversed the tumor suppressive roles of LARRPM in LUAD

**Fig. 5:**
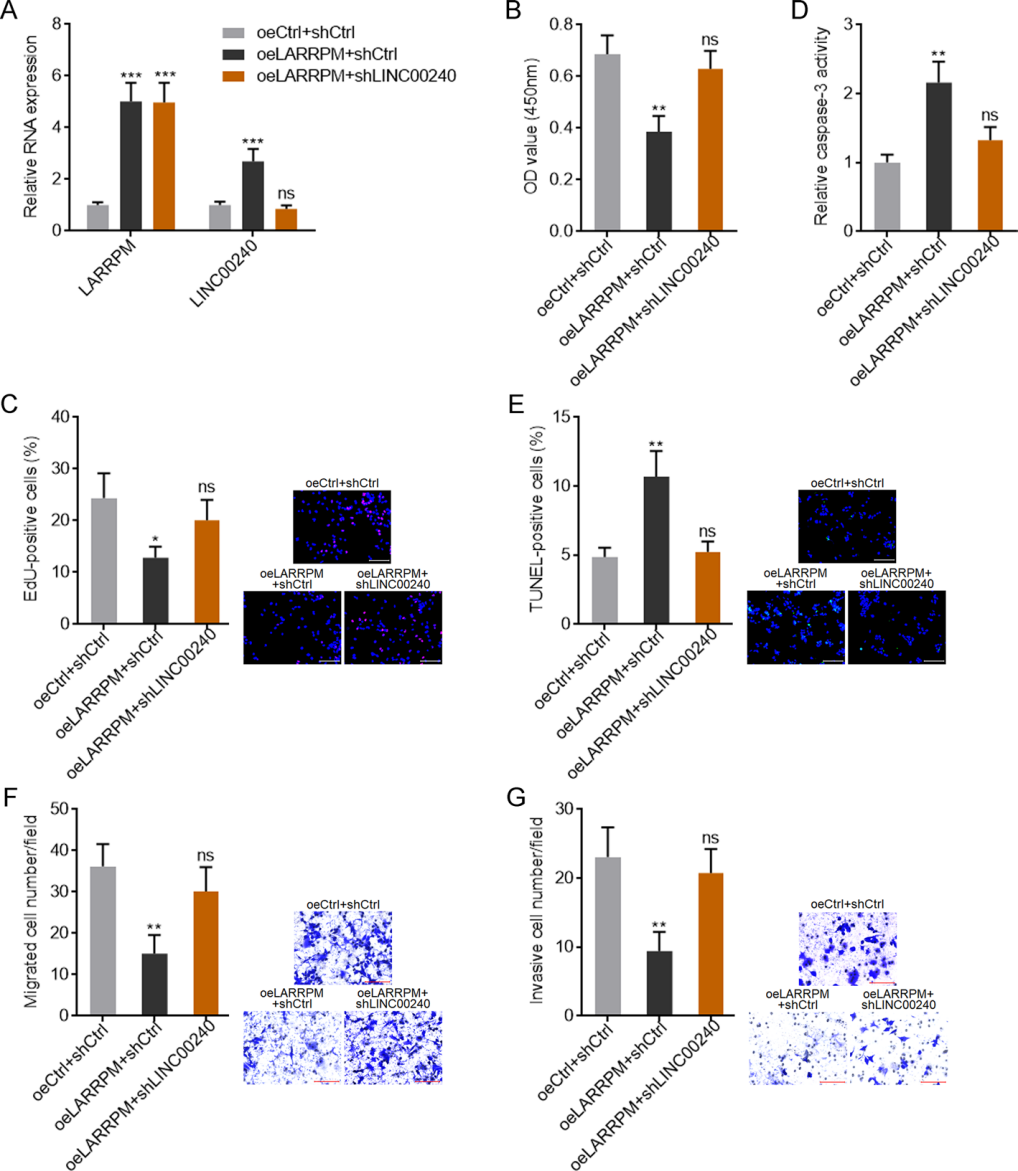
Depletion of the LINC00240 reversed the roles of LARRPM in proliferation, apoptosis, migration and invasion of LUAD cells. **a** LARRPM and LINC00240 expression in A549 cells with LARRPM overexpression; LINC00240 depletion was detected by qRT-PCR. **b**, **c** Cell proliferation of A549 cells with LARRPM overexpression and LINC00240 depletion was detected using the CCK-8 assay (**b**) and EdU incorporation assay (**c**). Scale bar: 100 µm. Red color indicates the EdU-positive cells. **d**, **e** Cell apoptosis of A549 cells with LARRPM overexpression and LINC00240 depletion was detected using the caspase-3 activity assay (**d**) and TUNEL assay (**e**). Scale bar: 100 µm. Green color indicates TUNEL-positive cells. **f, g** Cell migration and invasion of A549 cells with LARRPM overexpression and LINC00240 depletion were detected using the transwell migration assay (**f**) and transwell invasion assay (**g**). Scale bar: 100 µm. Results are shown as the mean ± SD based on three independent experiments. Asterisks indicate a significant difference at *P < 0.05, **P < 0.01, ***P < 0.001; ns, not significant, by one-way ANOVA followed by Dunnett's multiple comparisons test

To further confirm the roles of LINC00240 in LUAD, we constructed A549 cells with LINC00240 stable overexpression (Additional file 4: Figure S3A). CCK-8 and EdU incorporation assays showed that overexpression of LINC00240 repressed cell proliferation (Additional file 4: Figure S3B, C). The results of the caspase-3 activity and TUNEL assays showed that overexpression of LINC00240 promoted cell apoptosis (Additional file 4: Figure S3D, E), and the results of the transwell migration and invasion assays showed that ectopic expression of LINC00240 repressed cell migration and invasion (Additional file 4: Figure S3F, G). These findings suggested that consistent with LARRPM, LINC00240 also exerted tumor suppressive effects in LUAD. To investigate whether LINC00240 mediates the tumor suppressive roles of LARRPM in LUAD, we stably depleted LINC00240 expression in A549 cells with LARRPM stable overexpression (Fig. 5a). The CCK-8 and EdU incorporation assays revealed that depletion of LINC00240 attenuated the roles of LARRPM in repressing cell proliferation (Fig. 5b, c); the caspase-3 activity and TUNEL assays revealed that depletion of LINC00240 attenuated the roles of LARRPM in promoting cell apoptosis (Fig. 5e, e); and the transwell migration and invasion assays revealed that depletion of LINC00240 attenuated the roles of LARRPM in repressing cell migration and invasion (Fig. 5f, g). Take together, these findings suggested that LINC00240 at least partially mediated the tumor suppressive roles of LARRPM in LUAD.

### LARRPM repressed macrophages infiltration and M2 polarization

**Fig. 6:**
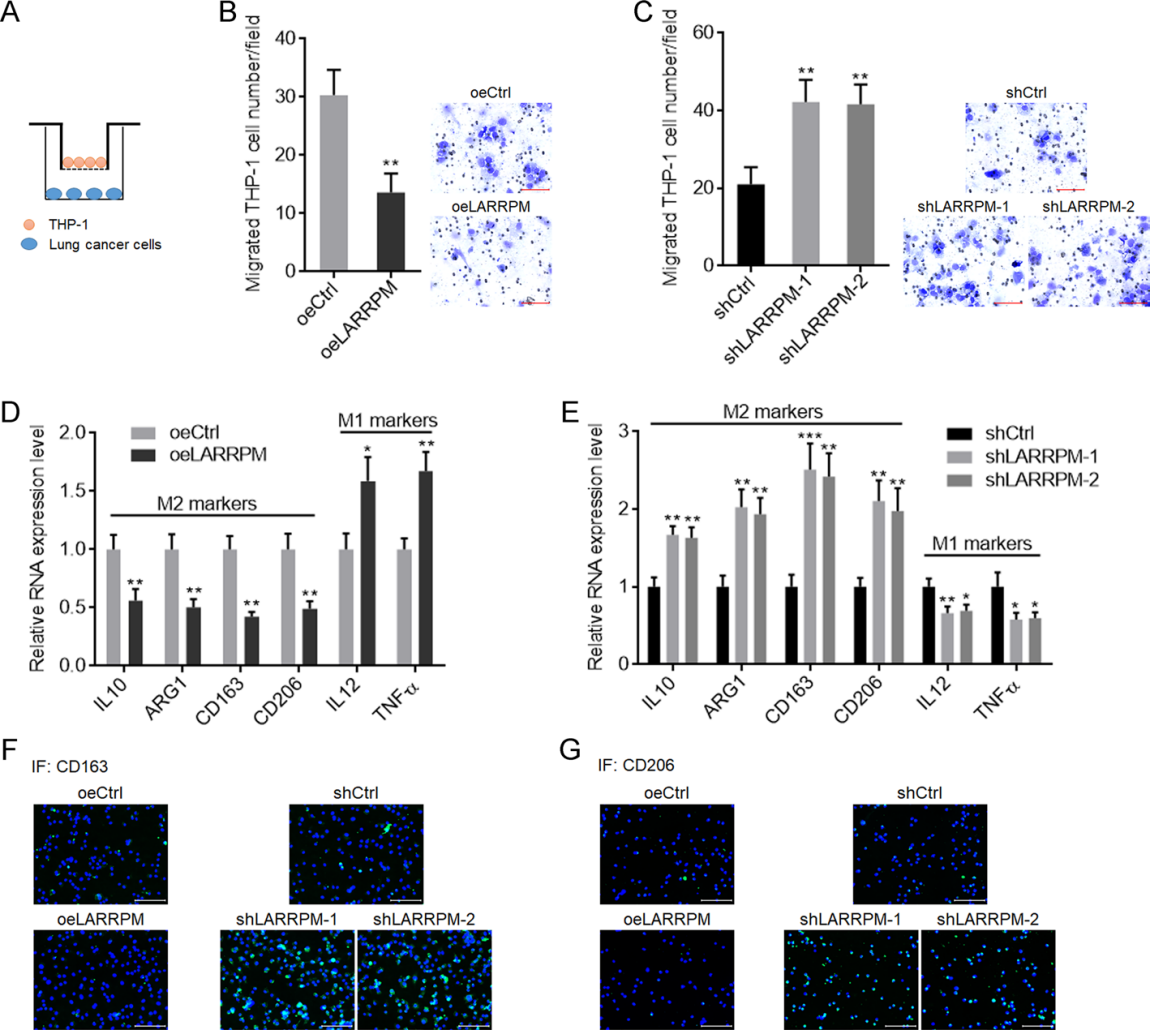
LARRPM repressed M2 macrophage infiltration. **a** Schematic diagram of in vitro co-culture system of phorbol-12-myristate-13-acetate (PMA)-stimulated human monocyte line THP-1 cells and LUAD cells. **b** THP-1 cells were subjected to transwell migration assays towards A549 cells with LARRPM overexpression or A549 control cells. Scale bar: 100 µm. c THP-1 cells were subjected to transwell migration assays towards HCC827 cells with LARRPM depletion or HCC827 control cells. Scale bar: 100 µm. **d** Expression of M1 and M2 polarization markers in THP-1 cells co-cultured with A649 cells with LARRPM overexpression or A549 control cells was detected by qRT-PCR. **e** Expression of M1 and M2 polarization markers in THP-1 cells co-cultured with HCC827 cells with LARRPM depleted or HCC827 control cells was detected by qRT-PCR. **f**, **g** CD163 (**f**) and CD206 (**g**) IF staining in THP-1 cells co-cultured with A549 cells with LARRPM overexpression or A549 control cells, or THP-1 cells co-cultured with HCC827 cells with LARRPM depleted or HCC827 control cells. Results are shown as the mean ± SD based on three independent experiments. Asterisks indicate a significant difference at *P < 0.05, **P < 0.01, ***P < 0.001, by Student’s *t*-test (**b**, **d**) or one-way ANOVA followed by Dunnett's multiple comparisons test (**c**, **e**).* ARG1* Arginase 1,* IL* interleukin,* TNF* tumor necrosis factor

Observing that LARRPM repressed macrophage infiltration in vivo, we further investigated the effects of LARRPM on macrophages by in vitro co-culture system (Fig. 6a). A549 cells with LARRPM overexpression inhibited macrophage migration compared with control A549 cells (Fig. 6b). HCC827 cells with LARRPM silencing promoted macrophage migration compared with control HCC827 cells (Fig. 6c). The expressions of M1 and M2 markers in macrophages after co-culture with LUAD cells were measured. It was noted that after co-culture with LARRPM overexpressed A549 cells, M2 markers in macrophages were decreased and M1 markers were increased, compared with those co-cultured with control A549 cells (Fig. 6d). Consistently, M2 markers were increased and M1 markers were decreased after co-culture with LARRPM silenced HCC827 cells, compared with those co-cultured with control HCC827 cells (Fig. 6e). IF staining of cell surface protein CD163 and CD206, which are classical M2 markers, revealed that both CD163 and CD206 levels were decreased after co-culture with A549 cells overexpressing LARRPM and increased after co-culture with LARRPM silenced HCC827 cells (Fig. 6f, g). These findings suggested that LARRPM repressed infiltration of macrophages and M2 polarization.

### LARRPM decreased infiltrated M2 macrophage via repressing the production of CSF1

**Fig. 7:**
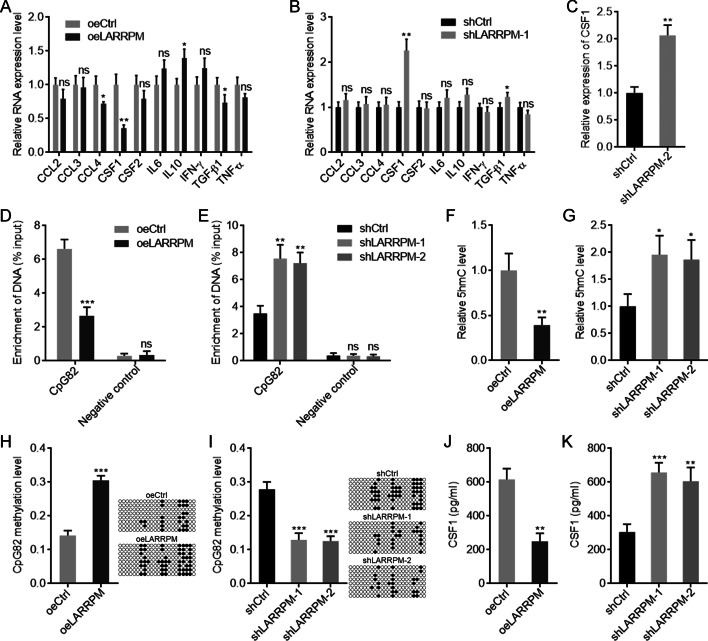
LARRPM repressed CSF1 expression via inducing DNA methylation. **a**, **b** The expression of cytokines related to macrophage recruitment and polarization in A549 cells with LARRPM overexpression (**a**) and HCC827 cells with LARRPM depletion (**b**) was detected by qRT-PCR. **c** The expression of CSF1 in another LARRPM stably depleted HCC827 clone was detected by qRT-PCR. **d**, **e** ChIP assays followed by qPCR were performed in A549 cells with LARRPM overexpression (**d**) and HCC827 cells with LARRPM depletion (**e**) to detect the DNAs (*LINC00240* promoter) bound by TET1. **f**, **g** 5hmC levels of the CpG island CpG82 from A549 cells with LARRPM overexpression (**f**) and HCC827 cells with LARRPM depletion (**g**) were measured using the EpiMark 5-hmC Analysis Kit. **h**, **i** DNA methylation levels of CpG82 from A549 cells with LARRPM overexpression (**h**) and HCC827 cells with LARRPM depletion (**i**) were measured using bisulfate DNA sequencing. **j**, **k** CSF1 secretion from A549 cells with LARRPM overexpression (**j**) and HCC827 cells with LARRPM depletion (**k**) was measured by enzyme-linked immunosorbent assay (ELISA). Results are shown as the mean ± SD based on three independent experiments. Asterisks indicate a significant different at *P < 0.05, **P < 0.01, ***P < 0.001, ns, not significant, by Student’s *t*-test (**a**–**d**, **f**, **h**, **j**) or one-way ANOVA followed by Dunnett's multiple comparisons test (**e**, **g**, **i**, **k**).* CCL2, -3, -4* CC chemokine family,* CSF1, CSF2* Colony Stimulating Factor 1,* IFN* interferon,* TGF* tumor growth factor

To dissect the mechanisms mediating the roles of LARRPM in M2 macrophage infiltration, we measured the expression of cytokines which were involved in macrophage recruitment and polarization [46, 47]. The results showed that CSF1 was the most significantly altered molecule after LARRPM overexpression or depletion (Fig. 7a, b), with its level decreasing after LARRPM overexpression and increasing after LARRPM silencing (Fig. 7a–c). Given that the promoter of *CSF1* was also occupied by the CpG island CpG82, we investigated whether LARRPM modulates the binding of TET1 to the *CSF1* promoter. Conversely to the effects of LARRPM on the *LINC00240* promoter, ectopic expression of LARRPM decreased the binding of TET1 to the *CSF1* promoter (Fig. 7d), while depletion of LARRPM increased the binding of TET1 to the *CSF1* promoter (Fig. 7e). In line with the binding of TET1 to the *CSF1* promoter, ectopic expression of LARRPM decreased the 5hmC level at the *CSF1* promoter, while depletion of LARRPM increased the 5hmC level (Fig. 7f, g). The DNA methylation level of CpG82 increased when LARRPM was overexpressed, and decreased when LARRPM was depleted (Fig. 7h, i). An ELISA showed that ectopic expression of LARRPM decreased CSF1 concentration in the supernatant, while silencing of LARRPM increased CSF1 concentration in the supernatant (Fig. 7j, k). The TCGA LUAD dataset showed a negative correlation between CSF1 and LARRPM expression in LUAD tissues (Additional file 5: Figure S4A). In our own cohort, a significant negative correlation between CSF1 and LARRPM expression was also found in LUAD tissues (Additional file 5: Figure S4B). Furthermore, LUAD tissues with high LARRPM expression had a reduced 5hmC level at the *CSF1* promoter compared with LUAD tissues with low LARRPM expression (Additional file 5: Figure S4C). LUAD tissues with high LARRPM expression had an increased DNA methylation level of CpG82 compared with LUAD tissues with low LARRPM expression (Additional file 5: Figure S4D). These findings supported the negative regulation of CSF1 by LARRPM via TET1-mediated DNA demethylation.

To assess whether CSF1 mediates the roles of LARRPM in repressing M2 macrophage infiltration, anti-CSF1 antibody was added to LUAD cells. In the presence of anti-CSF1, LARRPM overexpression in A549 cells did not regulate macrophage migration (Additional file 6: Figure S5A). Similarly, in the presence of anti-CSF1, LARRPM silencing in HCC827 cells also did not regulate macrophage migration (Additional file 6: Figure S5B). In the presence of anti-CSF1, LARRPM overexpression in A549 cells did not regulate the expressions of M1 and M2 markers in macrophages after co-culture (Additional file 6: Figure S5C). Similarly, in the presence of anti-CSF1, LARRPM silencing in HCC827 cells also not regulate the expressions of M1 and M2 markers in macrophages after co-culture (Additional file 6: Figure S5D). Collectively, these findings suggested that LARRPM repressed CSF1 production via increasing DNA methylation level of *CSF1* promoter. CSF1 was the critical mediator of the roles of LARRPM in repressing M2 macrophage infiltration.

### LARRPM attenuated the oncogenic roles of infiltrated M2 macrophages

**Fig. 8:**
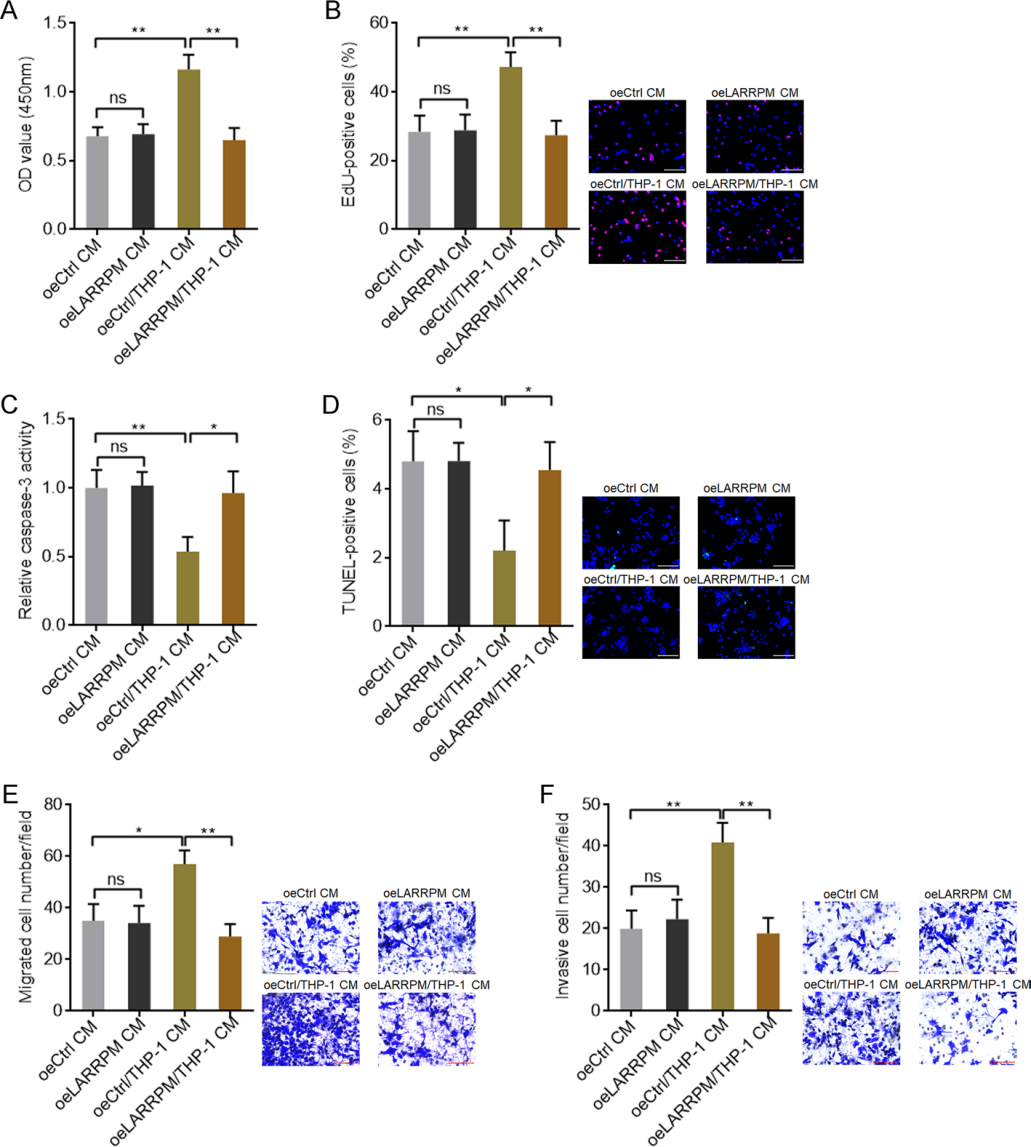
Ectopic expression of LARRPM in LUAD cells attenuated the oncogenic roles of infiltrated M2 macrophages. **a**–**f** CM was collected from co-culture of macrophages with LARRPM overexpressed or control A549 cells, or collected from only A549 cells with LARRPM overexpression or A549 control cells. **a**, **b** Cell proliferation of A549 cells treated with the CM was detected using the CCK-8 assay (**a**) and EdU incorporation assay (**b**). Scale bar: 100 µm. Red color indicates EdU-positive cells. **c**, **d** Cell apoptosis of A549 cells treated with the CM was detected using the caspase-3 activity assay (**c**) and TUNEL assay (**d**). Scale bar: 100 µm. Green color indicates TUNEL-positive cells. **e**, **f** Cell migration and invasion of A549 cells treated with the CM was detected using the transwell migration assay (**e**) and transwell invasion assay (**f**). Scale bar: 100 µm. Results are shown as the mean ± SD based on three independent experiments. Asterisks indicate a significant difference at *P < 0.05, **P < 0.01, ns, not significant, by Student’s *t*-test.* CM* Conditioned medium

We also looked at the roles of infiltrated M2 macrophages on LUAD cells. The conditioned medium (CM) was collected from co-culture of macrophages with A549 cells with LARRPM overexpression or control A549 cells. Next, mock A549 cells were treated with the the CM. CCK-8 and EdU incorporation assays revealed that macrophage/A549 co-culture CM promoted A549 cell proliferation compared with A549 CM (Fig. 8a, b). Ectopic expression of LARRPM repressed the increased proliferation of A549 cells induced by macrophages (Fig. 8a, b). The results of the caspase-3 activity and TUNEL assays showed that macrophage/A549 co-culture CM inhibited A549 cell apoptosis compared with A549 CM (Fig. 8c, d). Ectopic expression of LARRPM reversed the reduced apoptosis of A549 cells induced by macrophages (Fig. 8c, d). Transwell migration and invasion assays revealed that macrophage/A549 co-ulture CM promoted A549 cell migration and invasion compared with A549 CM (Fig. 8e, f). Ectopic expression of LARRPM repressed the increased migration and invasion of A549 cells induced by macrophages (Fig. 8e, f). CM was also collected from co-culture of macrophages with HCC827 with LARRPM depletion or control HCC827 cells, and this CM used to stimulate mock HCC827 cells. The CCK-8 and EdU incorporation assays revealed that macrophage/HCC827 co-culture CM promoted HCC827 cell proliferation compared with HCC827 CM (Additional file 7: Figure S6A, B). CM from the co-culture of macrophages with HCC827 cells with LARRPM depletion more strongly promoted HCC827 cell proliferation (Additional file 7: Figure S6A, B). The results of the caspase-3 activity and TUNEL assays showed that macrophage/HCC827 co-culture CM inhibited HCC827 cell apoptosis compared with HCC827 CM (Additional file 7: Figure S6C, D). CM from co-culture of macrophages with HCC827 cells with LARRPM more strongly inhibited HCC827 cell apoptosis (Additional file 7: Figure S6C, D). The transwell migration and invasion assays revealed that macrophage/HCC827 co-culture CM promoted HCC827 cell migration and invasion compared with HCC827 CM (Additional file 7: Figure S6E, F). CM from co-culture of macrophages with HCC827 cells with LARRPM depletion more strongly promoted HCC827 cell migration and invasion (Additional file 7: Figure S6E, F). These findings suggested that LUAD cells-educated M2 macrophages exerted oncogenic effects in LUAD, which were negatively modulated by LARRPM in LUAD cells.

## Discussion

We identified a novel lncRNA, LARRPM, which mediates the crosstalk between LUAD cells and macrophages. LARRPM is 954 nt long and has only one exon. It is predominantly distributed in the nucleus and has a polyA tail. In the present study, LARRPM was downregulated in LUAD tissues and cells compared with noncancerous lung tissues and bronchial epithelial cells. Low expression of LARRPM was correlated with large tumor size, local invasion, advanced TMN stage, and poor prognosis of patients with LUAD. Our data suggest that LARRPM is a potential prognostic biomarker for LUAD. The clinical relevance of LARRPM in other histological subtypes of lung cancer requires further study.

Our functional investigations showed that LARRPM repressed LUAD cell proliferation, migration and invasion, and promoted LUAD cell apoptosis in vitro. In vivo xenograft assays showed that LARRPM suppressed LUAD tumor growth and metastasis, repressed LUAD cell proliferation and induced LUAD cell apoptosis in vivo. Further, LARRPM was found to reduce the number of M2 macrophages in xenografts. In vitro dissections revealed that LARRPM repressed macrophage M2 polarization and migration. TAMs have been reported to have tumor promotive effects in many tumors [48–50]. In the present study, we also found that TAMs educated by LUAD cells promoted LUAD cell proliferation, migration and invasion, and repressed LUAD cell apoptosis. The oncogenic roles of TAMs decreased after co-culture with LUAD cells overexpressing LARRPM and increased after co-culture with LUAD cells with LARRPM depletion. Our findings suggest that LARRPM functions as a tumor repressor in LUAD through regulating both LUAD cells and TAMs.

Mechanistic dissections revealed that as a nuclear lncRNA, LARRPM bound to DNA demethylase TET1 and recruited TET1 to the promoter of its anti-sense strand gene *LINC00240*. Intriguingly, a CpG island was located at the promoter of *LINC00240*. Thus, LARRPM decreased the DNA methylation level of the *LINC00240* promoter, leading to the transcriptional activation of *LINC00240*. The positive correlation between the expression of LARRPM and LINC00240 in LUAD tissues supported the upregulation of LINC00240 by LARRPM in vivo. Functional rescue assays documented that LINC00240 was the critical mediator of the roles of LARRPM in LUAD cell proliferation, apoptosis, migration and invasion. The current study provides novel evidence for the roles of lncRNAs as epigenetic modulators. Many nuclear lncRNAs have been found to bind to epigenetic modification enzymes, such as histone methyltransferase, histone acetyltransferase, histone deacetylase and DNA methyltransferase [51–53]. Through binding to these epigenetic modification enzymes, lncRNAs change their genomic location, leading to alteration of the epigenetic modification status of target genes, and further the activation or repression of target gene transcription [51–53]. In this study, we further found that, with the exception of *LINC00240*, LARRPM also modulated the genomic binding of TET1 to the *CSF1* promoter. The *CSF1* promoter also had a CpG island. In contrast to the *LINC00240* promoter, LARRPM decreased the binding of TET1 to the *CSF1* promoter. Therefore, LARRPM increased the DNA methylation level of the *CSF1* promoter, leading to the transcriptional repression of *CSF1*. The negative correlation between LARRPM expression and CSF1 expression in LUAD tissues supports the downregulation of CSF1 by LARRPM in vivo. As a methylcytosine dioxygenase and a DNA demethylase, TET1 binds and regulates many genomic sites, such as the tumor suppressor TCF21 [54]. The potential influence of LARRPM on other TET1 targets needs further investigation.

CSF1, also known as macrophage colony stimulating factor (M-CSF), has been documented to induce M2 polarization, recruitment and survival in macrophages [47, 55]. In the present study, we identified CSF1 as a critical downstream target of LARRPM. LARRPM repressed *CSF1* transcription, leading to the decreasing of CSF1 secretion. Functional rescue assays using CSF1 blocking antibody documented that CSF1 was a critical mediator of the roles of LARRPM in macrophage polarization and recruitment. M2-polarized TAMs have been found to be correlated with poor survival in lung cancer [56]. Here we also found that macrophages educated by LUAD cells exerted oncogenic effects on LUAD cells. LARRPM overexpressed LUAD cells overexpression LARRPM repressed the oncogenic roles of macrophages after co-culture, which was consistent with the repressive roles of LARRPM on macrophage infiltration and M2 polarization. Our findings suggest that the LARRPM-CSF1 regulatory axis at least partially mediated the crosstalk between LUAD cells and macrophages.

## Conclusions

In summary, we identified LARRPM as a novel LUAD-related lncRNA. LARRPM was lowly expressed in LUAD, and its low expression was correlated with poor prognosis of patients with LUAD. LARRPM was observed to act as a tumor suppressor in LUAD through regulating both LUAD cells and macrophages. LARRPM repressed the malignant behaviors of LUAD cells via epigenetically upregulating LINC00240. LARRPM repressed macrophage infiltration and M2 polarization via epigenetically repressing CSF1. Our findings suggest LARRPM as a prognostic biomarker for LUAD, and enhancing LARRPM expression might be a novel strategy for LUAD treatment.

## Supplementary Information

**Additional file 1: Table S1.** Relationships between LARRPM expression and clinicopathological features in LUAD.

**Additional file 2: Figure S1.** Depletion of LARRPM promoted proliferation, repressed apoptosis and promoted migration and invasion of LUAD cells. **A** LARRPM expression in HCC827 cells with LARRPM stable depletion or control was detected by qRT-PCR. **B** Cell proliferation of HCC827 cells with LARRPM stable depletion or control was detected using CCK-8 assays. **C** Cell proliferation of HCC827 cells with LARRPM stable overexpression or control was detected using EdU incorporation assays. Scale bar: 100 µm. Red color indicates EdU-positive cells. **D** Cell apoptosis of HCC827 cells with LARRPM stable depletion or control was detected using caspase-3 activity assays. **E** Cell apoptosis of HCC827 cells with LARRPM stable depletion or control was detected using TUNEL assays. Scale bar: 100 µm. Green color indicates TUNEL-positive cells. **F** Cell migration of HCC827 cells with LARRPM stable depletion or control was detected using transwell migration assays. Scale bar: 100 µm. **G** Cell invasion of HCC827 cells with LARRPM stable depletion or control was detected using transwell invasion assays. Scale bar: 100 µm. Results are shown as mean ± SD based on three independent experiments. *P < 0.05, **P < 0.01, ***P < 0.001 by one-way ANOVA followed by Dunnett's multiple comparisons test.

**Additional file 3: Figure S2.** The correlation between LARRPM expression, 5hmC level at *LINC00240* promoter, CpG43 methylation level and LINC00240 expression in LUAD tissues. **A** Correlation between LINC00240 and LARRPM expression analysed using the TCGA LUAD data. *r* = 0.6164, *P* < 0.0001 by Spearman correlation analysis. **B** Correlation between LINC00240 and LARRPM expression analyzed in our LUAD cohort. *r* = 0.5521, *P* < 0.0001 by Spearman correlation analysis. **C** 5hmC levels of CpG43 from 20 LUAD tissues were measured using the EpiMark 5-hmC Analysis Kit. Median LARRPM expression level was used as cut-off. *P* = 0.0094 by Mann–Whitney test. **D** DNA methylation levels of CpG43 from 20 LUAD tissues were measured using bisulfate DNA sequencing. Median LARRPM expression level was used as cut-off. *P* = 0.0015 by Mann–Whitney test.

**Additional file 4: Figure S3.** LINC00240 repressed proliferation, induced apoptosis and inhibited migration and invasion of LUAD cells. **A** LINC00240 expression in A549 cells with LINC00240 stable overexpression or control was detected by qRT-PCR. **B** Cell proliferation of A549 cells with LINC00240 stable overexpression or control was detected using CCK-8 assays. **C** Cell proliferation of A549 cells with LINC00240 stable overexpression or control was detected using EdU incorporation assays. Scale bar: 100 µm. Red color indicates EdU-positive cells. **D** Cell apoptosis of A549 cells with LINC00240 stable overexpression or control was detected using caspase-3 activity assays. **E** Cell apoptosis of A549 cells with LINC00240 stable overexpression or control was detected using TUNEL assays. Scale bar: 100 µm. Green color indicates TUNEL-positive cells. **F** Cell migration of A549 cells with LINC00240 stable overexpression or control was detected using transwell migration assays. Scale bar: 100 µm. **G** Cell invasion of A549 cells with LINC00240 stable overexpression or control was detected using transwell invasion assays. Scale bar: 100 µm. Results are shown as mean ± SD based on three independent experiments. *P < 0.05, **P < 0.01 by Student’s *t*-test.

**Additional file 5: Figure S4.** The correlation between LARRPM expression, 5hmC level at *CSF1* promoter, CpG82 methylation level and CSF1 expression in LUAD tissues. **A** Correlation between CSF1 and LARRPM expression analysed using the TCGA LUAD data. *r* = − 0.3069, *P* < 0.0001 by Spearman correlation analysis. **B** Correlation between CSF1 and LARRPM expression analysed in our LUAD cohort. *r* = − 0.5774, *P* < 0.0001 by Spearman correlation analysis. **C** 5hmC levels of CpG82 from 20 LUAD tissues were measured using the EpiMark 5-hmC Analysis Kit. Median LARRPM expression level was used as cut-off. *P* = 0.0245 by Mann–Whitney test. **D** DNA methylation levels of CpG82 from 20 LUAD tissues were measured using bisulfate DNA sequencing. Median LARRPM expression level was used as cut-off. *P* = 0.0288 by Mann–Whitney test.

**Additional file 6: Fig. S5.** Anti-CSF1 antibody abolished the effects of LARRPM on M2 macrophage infiltration. **A** THP-1 cells were subjected to transwell migration assays towards A549 cells with LARRPM overexpression or A549 control cells treated with anti-CSF1. Scale bar: 100 µm. **B** THP-1 cells were subjected to transwell migration assays towards HCC827 cells with LARRPM depletion or HCC827 control treated with anti-CSF1. Scale bar: 100 µm. **C** M1 and M2 polarization markers expression in THP-1 cells co-cultured with A549 cells with LARRPM overexpression or control A549 cells treated with anti-CSF1 were detected by qRT-PCR. **D** Expression of M1 and M2 polarization markers in THP-1 cells co-cultured with HCC827 cells with LARRPM depletion or control HCC827 cells treated with anti-CSF1 were detected by qRT-PCR. Results are shown as the mean ± SD based on three independent experiments. ns: Not significant by Student’s *t*-test.

**Additional file 7: Fig. S6.** Depletion of LARRPM in LUAD cells enhanced the oncogenic roles of infiltrated M2 macrophages. **A–F** Conditioned medium (CM) was collected from co-culture of macrophages with HCC827 cells with LARRPM depletion or control HCC827 cells, or collected from only HCC827 cells with LARRPM depletion or control HCC827 cells. **A** Cell proliferation of HCC827 cells treated with the CM was detected using CCK-8 assays. **B** Cell proliferation of HCC827 cells treated with the CM was detected using EdU incorporation assays. Scale bar: 100 µm. Red color indicates EdU-positive cells. **C** Cell apoptosis of HCC827 cells treated with the CM was detected using caspase-3 activity assays. **D** Cell apoptosis of HCC827 cells treated with the CM was detected using TUNEL assays. Scale bar: 100 µm. Green color indicates TUNEL-positive cells. **E** Cell migration of HCC827 cells treated with the CM was detected using transwell migration assays. Scale bar: 100 µm. **F** Cell invasion of HCC827 cells treated with the CM was detected using transwell invasion assays. Scale bar: 100 µm. Results are shown as the mean ± SD based on three independent experiments. *P < 0.05, **P < 0.01, ns, not significant, by Student’s *t*-test (comparison between shCtrl CM and shCtrl/THP-1 CM groups) or one-way ANOVA followed by Dunnett's multiple comparisons test (comparison between shCtrl CM, shLARRPM-1 CM, and shLARRPM-2 CM groups, and between shCtrl/THP-1 CM, shLARRPM-1/THP-1 CM, and shLARRPM-2/THP-1 CM groups).

## Data Availability

The data presented in this study are available on reasonable request from the corresponding author.

## Abbreviations

CCK-8: Cell Counting Kit-8
cDNA: Complementary DNA
ChIP: Chromatin immunoprecipitation
EdU: 5-Ethynyl-2′-deoxyuridine
ELISA: Enzyme linked immunosorbent assay
FBS: Fetal bovine serum
HE: Hematoxylin and eosin
IF: Immunofluorescence
IHC: Immunohistochemistry
lncRNA: Long non-coding RNA
LARRPM: LINC00240 antisense RNA regulating promoter methylation
LUAD: Lung adenocarcinoma
NCBI: National Center for Biotechnology Information
ncRNA: Non-coding RNA
PMA: Phorbol-12-myristate-13-acetate
qPCR: Quantitative PCR
RIP: RNA immunoprecipitation
RT: Reverse transcription
TAM: Tumor-associated macrophage
TME: Tumor microenvironment
TUNEL: Terminal deoxynucleotidyl transferase (TdT)-mediated dUTP nick end labeling
