# Supplementary material for: LARRPM restricts lung adenocarcinoma progression and M2 macrophage polarization through epigenetically regulating LINC00240 and CSF1
Source: Cell Mol Biol Lett. 2022 Oct 11;27:91. doi: 10.1186/s11658-022-00376-y (PMC9552444; doi:10.1186/s11658-022-00376-y)
Supplement: Supplementary file 1 — Additional file 1: Table S1. Relationships between LARRPM expression and clinicopathological features in LUAD. [file 11658_2022_376_MOESM1_ESM.docx]

**Table S1** **Relationships between LARRPM expression and clinicopathological features in LUAD.**

| Parameters | No. of patients | LARRPM | | P*-*value |
| --- | --- | --- | --- | --- |
|  |  | low | high |  |
| Age |  |  |  | 0.626 |
| ≥ 60 | 42 | 22 | 20 |  |
| < 60 | 28 | 13 | 15 |  |
| Gender |  |  |  | 0.231 |
| Male | 33 | 14 | 19 |  |
| Female | 37 | 21 | 16 |  |
| Tumor size |  |  |  | 0.027 |
| ≤ 3cm | 27 | 9 | 18 |  |
| > 3cm | 43 | 26 | 17 |  |
| Local invasion |  |  |  | 0.019 |
| T1 | 22 | 6 | 16 |  |
| T2 | 39 | 22 | 17 |  |
| T3+T4 | 9 | 7 | 2 |  |
| Lymphatic metastasis |  |  |  | 0.089 |
| Negative | 41 | 17 | 24 |  |
| Positive | 29 | 18 | 11 |  |
| TNM stage |  |  |  | 0.031 |
| I | 30 | 10 | 20 |  |
| II | 31 | 18 | 13 |  |
| III-IV | 9 | 7 | 2 |  |

P values were calculated by Pearson chi-square test.
