# Supplementary figures and images for: LARRPM restricts lung adenocarcinoma progression and M2 macrophage polarization through epigenetically regulating LINC00240 and CSF1
Source: Cell Mol Biol Lett. 2022 Oct 11;27:91. doi: 10.1186/s11658-022-00376-y (PMC9552444; doi:10.1186/s11658-022-00376-y)

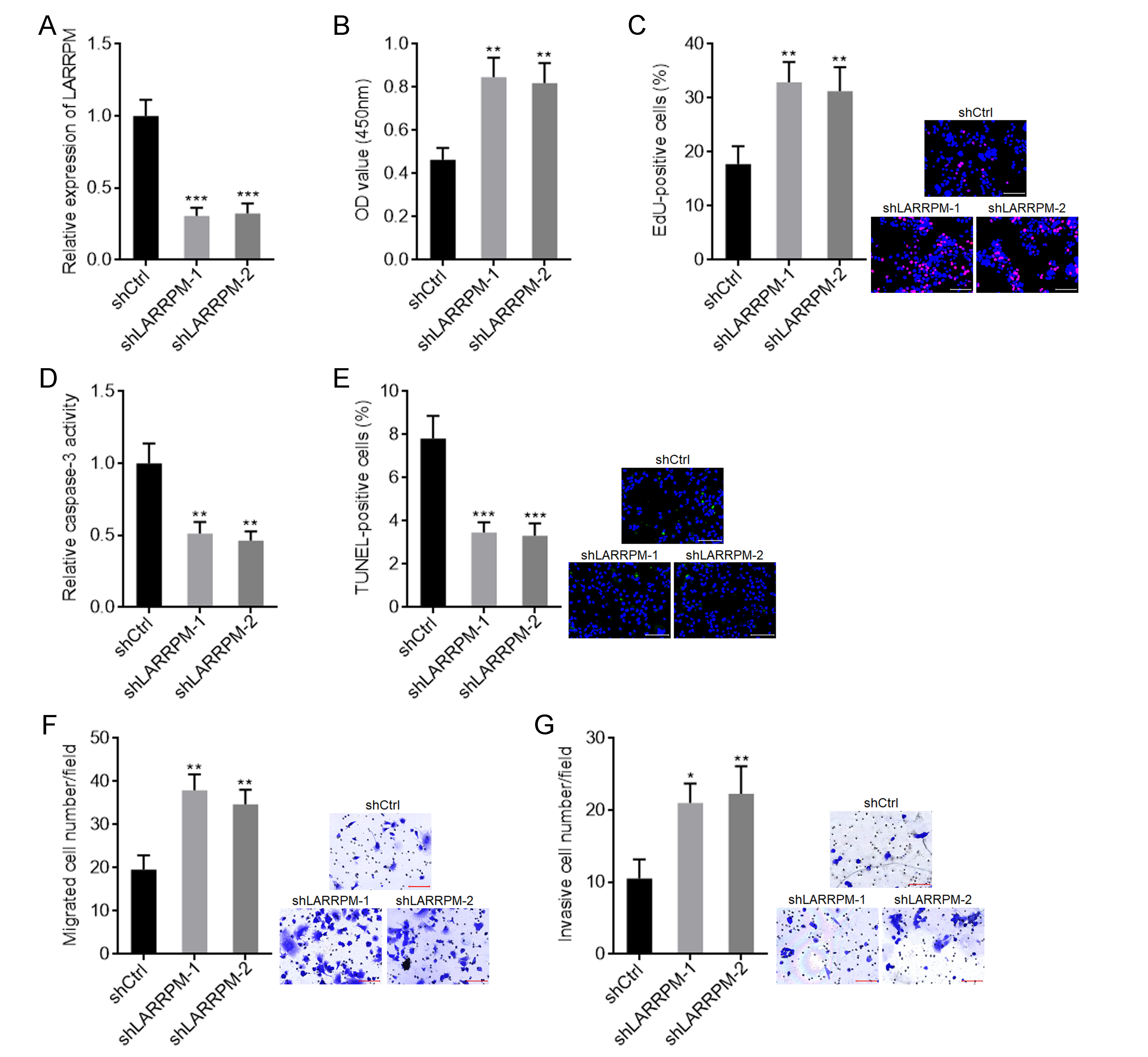

Supplement: Supplementary file 2 — Additional file 2: Figure S1. Depletion of LARRPM promoted proliferation, repressed apoptosis and promoted migration and invasion of LUAD cells. A LARRPM expression in HCC827 cells with LARRPM stable depletion or control was detected by qRT-PCR. B Cell proliferation of HCC827 cells with LARRPM stable depletion or control was detected using CCK-8 assays. C Cell proliferation of HCC827 cells with LARRPM stable overexpression or control was detected using EdU incorporation assays. Scale bar: 100 µm. Red color indicates EdU-positive cells. D Cell apoptosis of HCC827 cells with LARRPM stable depletion or control was detected using caspase-3 activity assays. E Cell apoptosis of HCC827 cells with LARRPM stable depletion or control was detected using TUNEL assays. Scale bar: 100 µm. Green color indicates TUNEL-positive cells. F Cell migration of HCC827 cells with LARRPM stable depletion or control was detected using transwell migration assays. Scale bar: 100 µm. G Cell invasion of HCC827 cells with LARRPM stable depletion or control was detected using transwell invasion assays. Scale bar: 100 µm. Results are shown as mean ± SD based on three independent experiments. *P < 0.05, **P < 0.01, ***P < 0.001 by one-way ANOVA followed by Dunnett's multiple comparisons test. [file 11658_2022_376_MOESM2_ESM.tif]

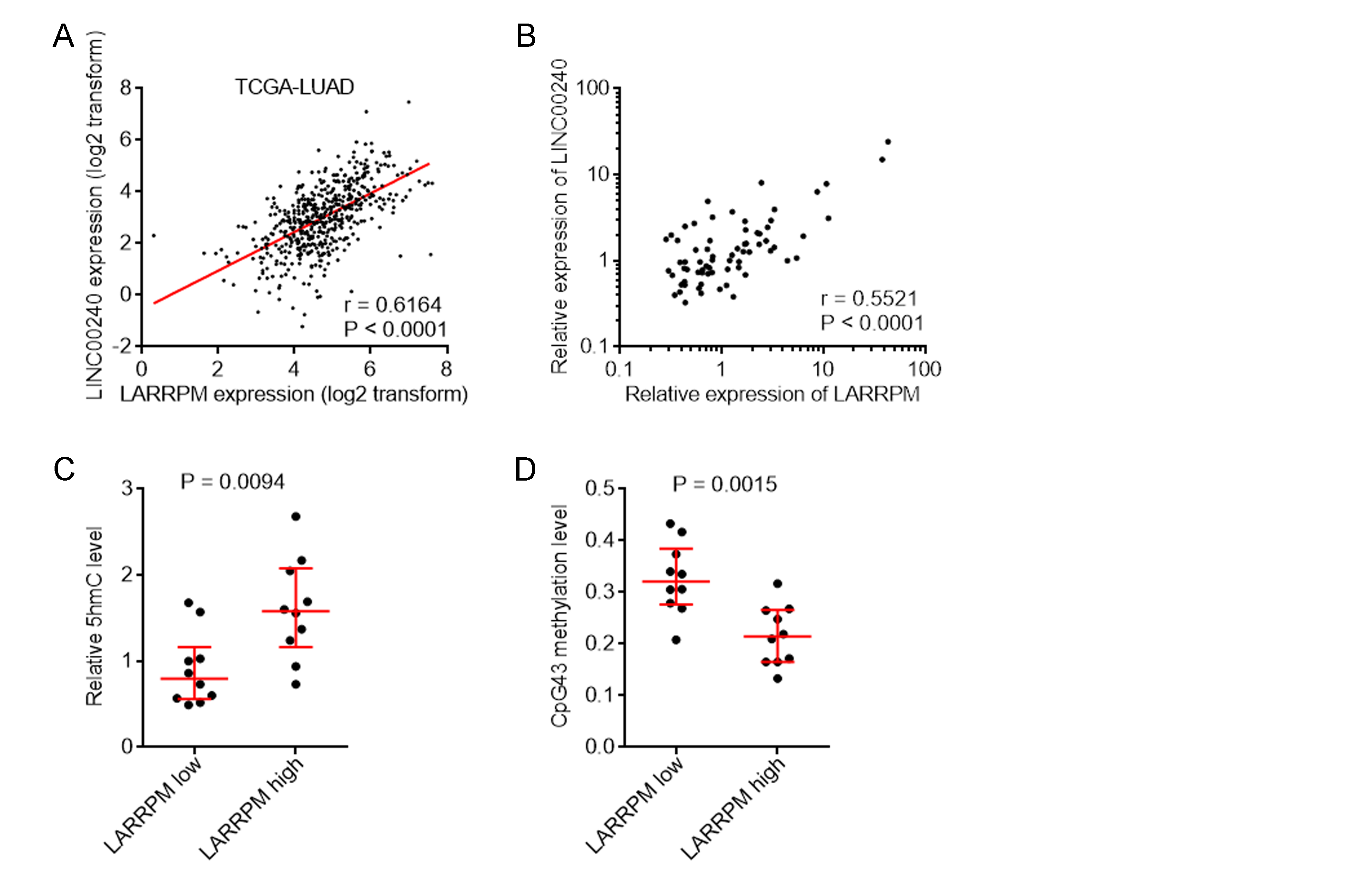

Supplement: Supplementary file 3 — Additional file 3: Figure S2. The correlation between LARRPM expression, 5hmC level at LINC00240 promoter, CpG43 methylation level and LINC00240 expression in LUAD tissues. A Correlation between LINC00240 and LARRPM expression analysed using the TCGA LUAD data. r = 0.6164, P < 0.0001 by Spearman correlation analysis. B Correlation between LINC00240 and LARRPM expression analyzed in our LUAD cohort. r = 0.5521, P < 0.0001 by Spearman correlation analysis. C 5hmC levels of CpG43 from 20 LUAD tissues were measured using the EpiMark 5-hmC Analysis Kit. Median LARRPM expression level was used as cut-off. P = 0.0094 by Mann–Whitney test. D DNA methylation levels of CpG43 from 20 LUAD tissues were measured using bisulfate DNA sequencing. Median LARRPM expression level was used as cut-off. P = 0.0015 by Mann–Whitney test. [file 11658_2022_376_MOESM3_ESM.tif]

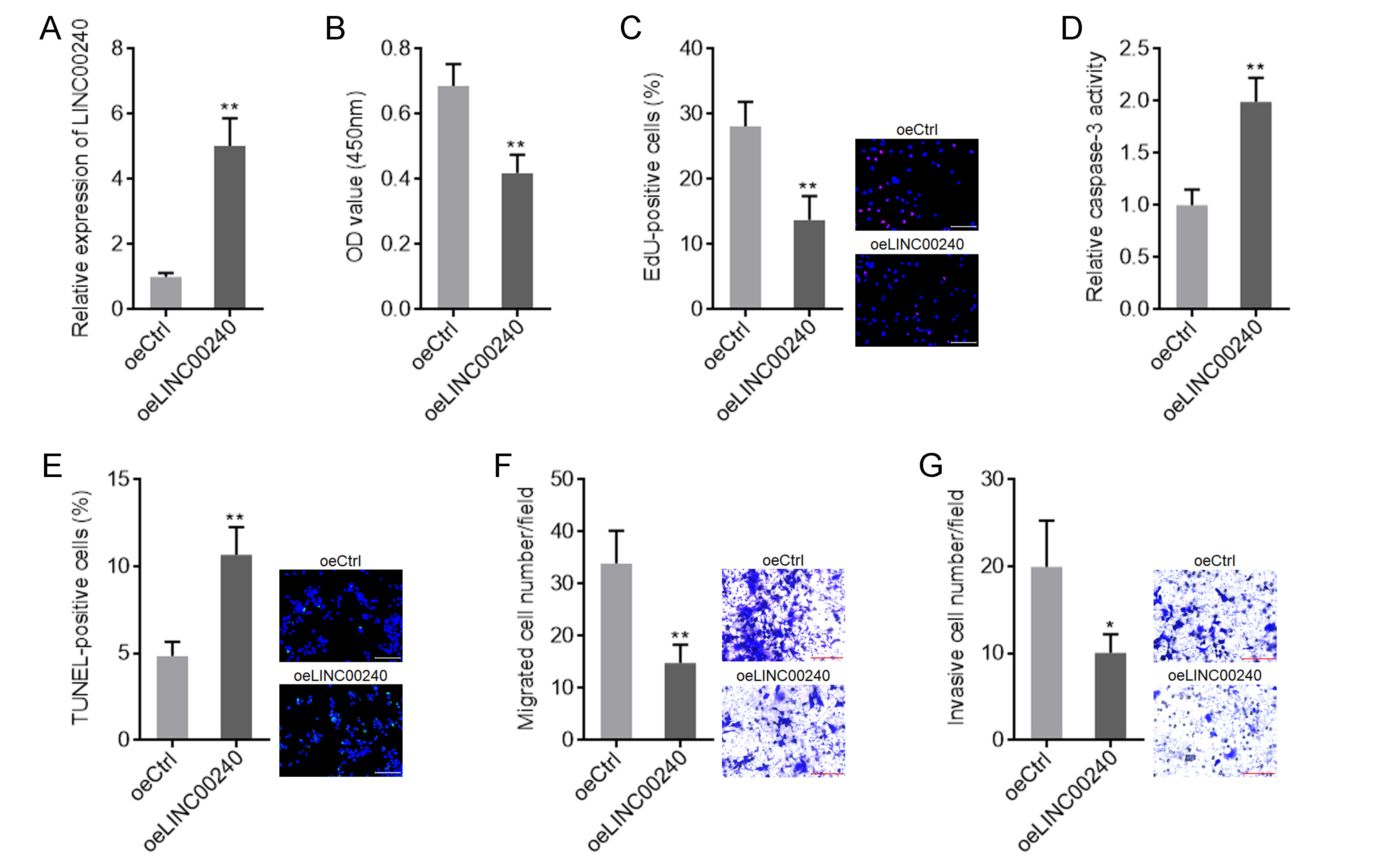

Supplement: Supplementary file 4 — Additional file 4: Figure S3. LINC00240 repressed proliferation, induced apoptosis and inhibited migration and invasion of LUAD cells. A LINC00240 expression in A549 cells with LINC00240 stable overexpression or control was detected by qRT-PCR. B Cell proliferation of A549 cells with LINC00240 stable overexpression or control was detected using CCK-8 assays. C Cell proliferation of A549 cells with LINC00240 stable overexpression or control was detected using EdU incorporation assays. Scale bar: 100 µm. Red color indicates EdU-positive cells. D Cell apoptosis of A549 cells with LINC00240 stable overexpression or control was detected using caspase-3 activity assays. E Cell apoptosis of A549 cells with LINC00240 stable overexpression or control was detected using TUNEL assays. Scale bar: 100 µm. Green color indicates TUNEL-positive cells. F Cell migration of A549 cells with LINC00240 stable overexpression or control was detected using transwell migration assays. Scale bar: 100 µm. G Cell invasion of A549 cells with LINC00240 stable overexpression or control was detected using transwell invasion assays. Scale bar: 100 µm. Results are shown as mean ± SD based on three independent experiments. *P < 0.05, **P < 0.01 by Student’s t-test. [file 11658_2022_376_MOESM4_ESM.tif]

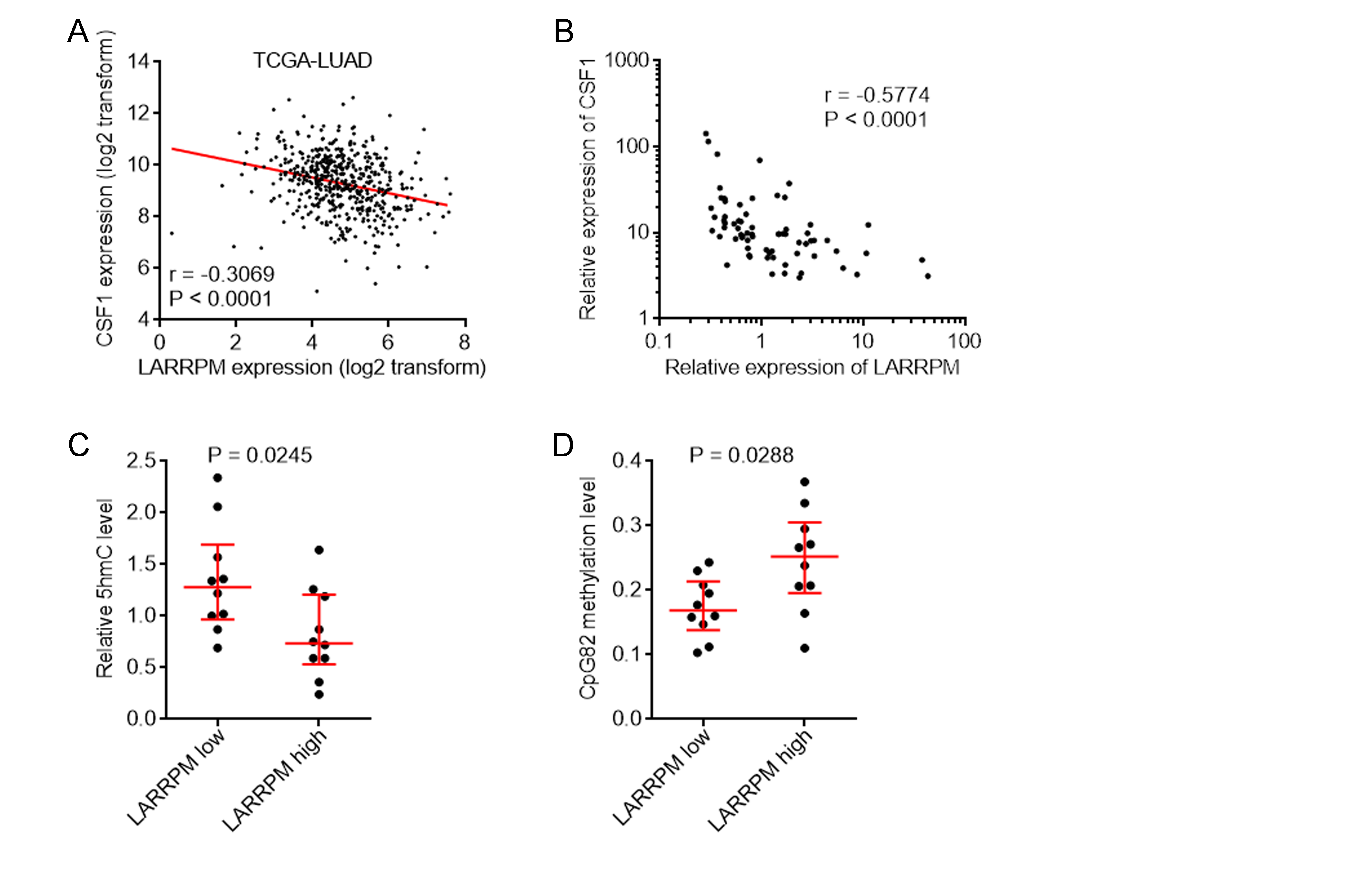

Supplement: Supplementary file 5 — Additional file 5: Figure S4. The correlation between LARRPM expression, 5hmC level at CSF1 promoter, CpG82 methylation level and CSF1 expression in LUAD tissues. A Correlation between CSF1 and LARRPM expression analysed using the TCGA LUAD data. r = − 0.3069, P < 0.0001 by Spearman correlation analysis. B Correlation between CSF1 and LARRPM expression analysed in our LUAD cohort. r = − 0.5774, P < 0.0001 by Spearman correlation analysis. C 5hmC levels of CpG82 from 20 LUAD tissues were measured using the EpiMark 5-hmC Analysis Kit. Median LARRPM expression level was used as cut-off. P = 0.0245 by Mann–Whitney test. D DNA methylation levels of CpG82 from 20 LUAD tissues were measured using bisulfate DNA sequencing. Median LARRPM expression level was used as cut-off. P = 0.0288 by Mann–Whitney test. [file 11658_2022_376_MOESM5_ESM.tif]

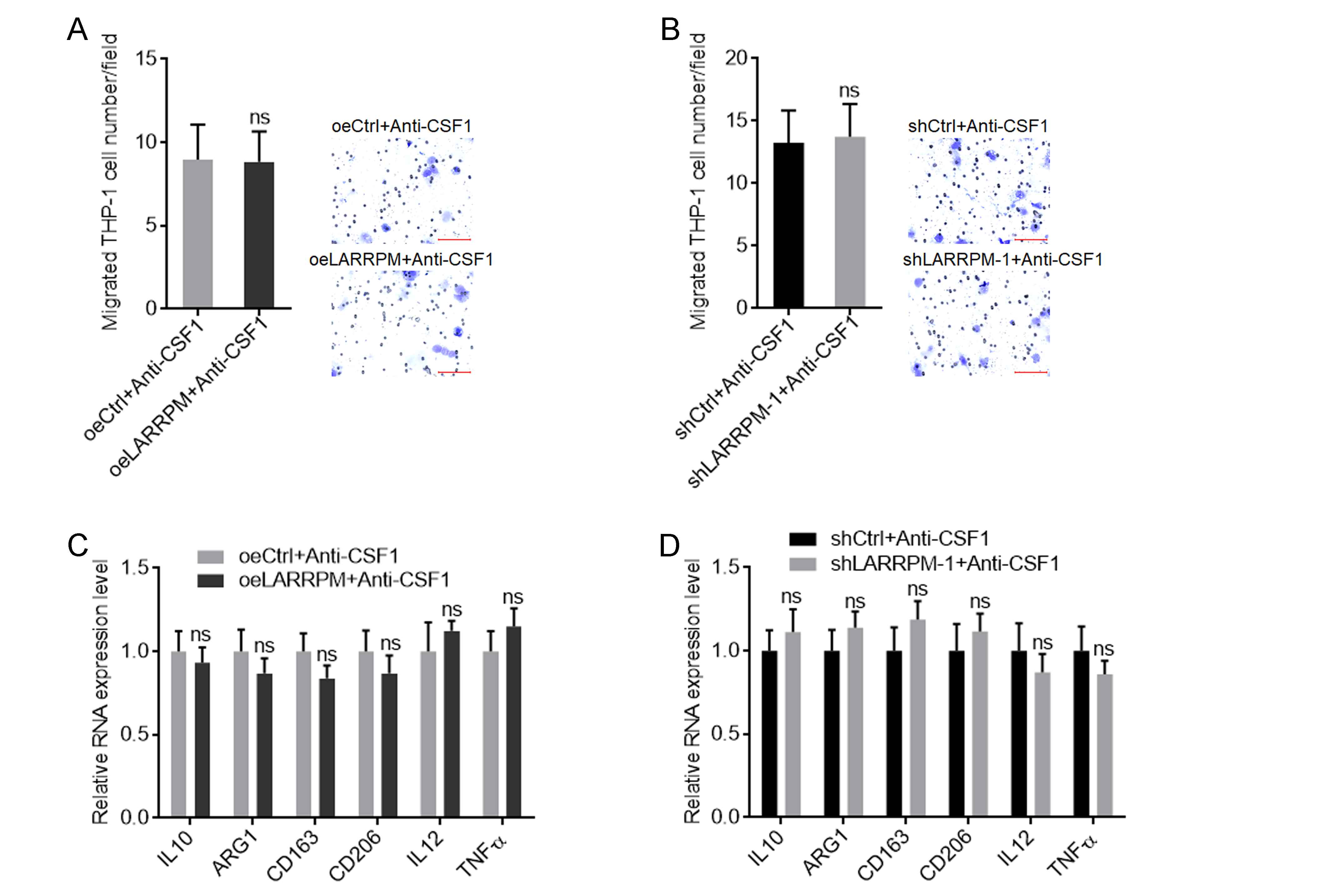

Supplement: Supplementary file 6 — Additional file 6: Fig. S5. Anti-CSF1 antibody abolished the effects of LARRPM on M2 macrophage infiltration. A THP-1 cells were subjected to transwell migration assays towards A549 cells with LARRPM overexpression or A549 control cells treated with anti-CSF1. Scale bar: 100 µm. B THP-1 cells were subjected to transwell migration assays towards HCC827 cells with LARRPM depletion or HCC827 control treated with anti-CSF1. Scale bar: 100 µm. C M1 and M2 polarization markers expression in THP-1 cells co-cultured with A549 cells with LARRPM overexpression or control A549 cells treated with anti-CSF1 were detected by qRT-PCR. D Expression of M1 and M2 polarization markers in THP-1 cells co-cultured with HCC827 cells with LARRPM depletion or control HCC827 cells treated with anti-CSF1 were detected by qRT-PCR. Results are shown as the mean ± SD based on three independent experiments. ns: Not significant by Student’s t-test. [file 11658_2022_376_MOESM6_ESM.tif]

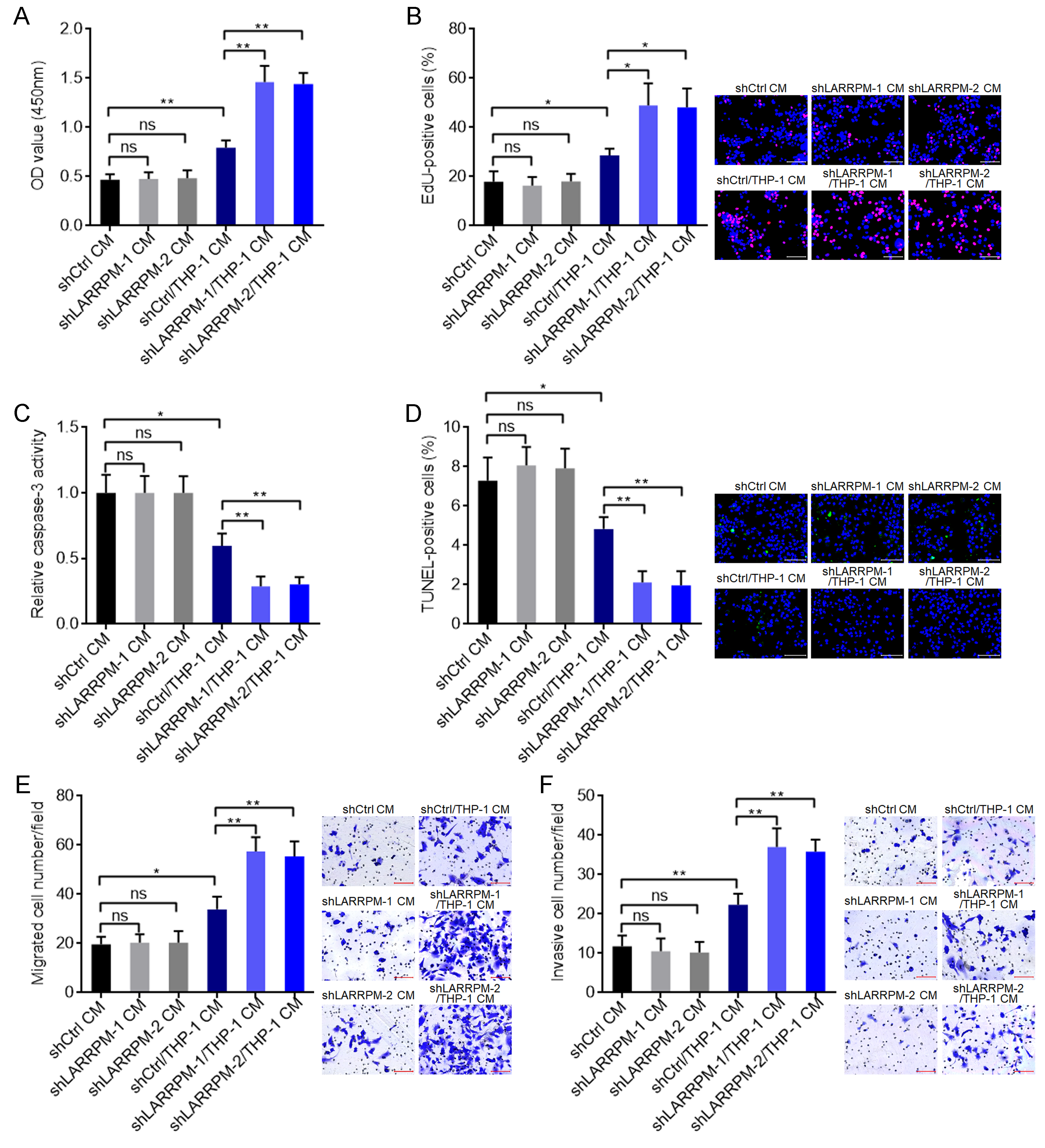

Supplement: Supplementary file 7 — Additional file 7: Fig. S6. Depletion of LARRPM in LUAD cells enhanced the oncogenic roles of infiltrated M2 macrophages. A–F Conditioned medium (CM) was collected from co-culture of macrophages with HCC827 cells with LARRPM depletion or control HCC827 cells, or collected from only HCC827 cells with LARRPM depletion or control HCC827 cells. A Cell proliferation of HCC827 cells treated with the CM was detected using CCK-8 assays. B Cell proliferation of HCC827 cells treated with the CM was detected using EdU incorporation assays. Scale bar: 100 µm. Red color indicates EdU-positive cells. C Cell apoptosis of HCC827 cells treated with the CM was detected using caspase-3 activity assays. D Cell apoptosis of HCC827 cells treated with the CM was detected using TUNEL assays. Scale bar: 100 µm. Green color indicates TUNEL-positive cells. E Cell migration of HCC827 cells treated with the CM was detected using transwell migration assays. Scale bar: 100 µm. F Cell invasion of HCC827 cells treated with the CM was detected using transwell invasion assays. Scale bar: 100 µm. Results are shown as the mean ± SD based on three independent experiments. *P < 0.05, **P < 0.01, ns, not significant, by Student’s t-test (comparison between shCtrl CM and shCtrl/THP-1 CM groups) or one-way ANOVA followed by Dunnett's multiple comparisons test (comparison between shCtrl CM, shLARRPM-1 CM, and shLARRPM-2 CM groups, and between shCtrl/THP-1 CM, shLARRPM-1/THP-1 CM, and shLARRPM-2/THP-1 CM groups). [file 11658_2022_376_MOESM7_ESM.tif]
